# PacBio full-length transcriptome of wild apple (*Malus sieversii*) provides insights into canker disease dynamic response

**DOI:** 10.1186/s12864-021-07366-y

**Published:** 2021-01-14

**Authors:** Xiaojie Liu, Xiaoshuang Li, Xuejing Wen, Yan Zhang, Yu Ding, Yiheng Zhang, Bei Gao, Daoyuan Zhang

**Affiliations:** 1 State Key Laboratory of Desert and Oasis Ecology, Xinjiang Institute of Ecology and Geography, Chinese Academy of Sciences, Urumqi, China; 2 University of Chinese Academy of Sciences, Beijing, China; 3 Turpan Eremophytes Botanical Garden, Chinese Academy of Sciences, Turpan, China; 4 Nanjing Forestry University, Nanjing, China

**Keywords:** *Malus sieversii*, Disease response, Jasmonic acid, Salicylic acid, PacBio Iso-Seq, Transcription factor

## Abstract

**Background:**

*Valsa* canker is a serious disease in the stem of *Malus sieversii*, caused by *Valsa mali*. However, little is known about the global response mechanism in *M. sieversii* to *V. mali* infection.

**Results:**

Phytohormone jasmonic acid (JA) and salicylic acid (SA) profiles and transcriptome analysis were used to elaborate on the dynamic response mechanism. We determined that the JA was initially produced to respond to the necrotrophic pathogen *V. mali* infection at the early response stage, then get synergistically transduced with SA to respond at the late response stage. Furthermore, we adopted Pacific Biosciences (PacBio) full-length sequencing to identify differentially expressed transcripts (DETs) during the canker response stage. We obtained 52,538 full-length transcripts, of which 8139 were DETs. Total 1336 lncRNAs, 23,737 alternative polyadenylation (APA) sites and 3780 putative transcription factors (TFs) were identified. Additionally, functional annotation analysis of DETs indicated that the wild apple response to the infection of *V. mali* involves plant-pathogen interaction, plant hormone signal transduction, flavonoid biosynthesis, and phenylpropanoid biosynthesis. The co-expression network of the differentially expressed TFs revealed 264 candidate TF transcripts. Among these candidates, the WRKY family was the most abundant. The *MsWRKY7* and *MsWRKY33* were highly correlated at the early response stage, and *MsWRKY6*, *MsWRKY7*, *MsWRKY19*, *MsWRKY33*, *MsWRKY40*, *MsWRKY45*, *MsWRKY51*, *MsWRKY61*, *MsWRKY75* were highly correlated at the late stage.

**Conclusions:**

The full-length transcriptomic analysis revealed a series of immune responsive events in *M. sieversii* in response to *V. mali* infection. The phytohormone signal pathway regulatory played an important role in the response stage. Additionally, the enriched disease resistance pathways and differentially expressed TFs dynamics collectively contributed to the immune response. This study provides valuable insights into a dynamic response in *M. sieversii* upon the necrotrophic pathogen *V. mali* infection, facilitates understanding of response mechanisms to canker disease for apple, and provides supports in the identification of potential resistance genes in *M. sieversii*.

**Supplementary Information:**

The online version contains supplementary material available at 10.1186/s12864-021-07366-y.

## Background

Wild apple (*Malus sieversii*) is widely distributed in the Tianshan Wild Fruit Forest area of Xinjiang, China. It is an ancestor of cultivated apple (*Malus domestica*) distributed in Central Asia to West Europe along the Silk Road [1] and is an isolated ecotype with a homogeneous genetic background that holds the underlying potential for the germplasm improvement of future apple [2]. However, the area of the Wild Fruit Forest in Xinjiang was dramatically reduced partly due to the *M. sieversii* was being attacked by the canker disease caused by necrotrophic pathogen *Valsa mali* and resulting apple tree condition weakening [3, 4]. Understanding the molecular mechanism of apple response to *V. mali* infection is important for gene utilization and apple protection. Yin et al. reported that 2713 genes in *M. domestica* were significantly up-regulated during *V. mali* infection through Illumina sequencing analysis, and SA/JA signaling pathways were mainly phytohormone pathways of apple response to the pathogen [5]. MdUGT88F1-mediated phloridzin biosynthesis plays a negative regulatory role in *Valsa* canker resistance [6]. However, in wild apple *M. sieversii,* little is known regarding the integral molecular mechanisms underlying the response to the infection of *V. mali*.

Phytohormone salicylic acid (SA), jasmonic acid (JA) and ethylene (ET) play major roles in regulating plant defense response against various pathogens [7]. SA is normally involved in the activation of defense response against biotrophic and hemibiotrophic pathogens [8], whereas JA and ET are responsible for host immunity to necrotrophic pathogens through the regulation of transcriptional activators and repressors of the ET and JA pathways [9, 10]. SA and JA hormone pathways are in an antagonistic relationship, and Non-Expressor of Pathogenesis-Related (PR) genes1 (NPR1) is the central regulator in the antagonistic crosstalk [7, 11]. Transcription factor WRKY70 is a key component maintaining the antagonistic relationship between the two hormones, which WRKY70 is activated by SA and inhibited by JA [12, 13].

Numerous plant transcriptional factors (TF) families genes have been identified that could be prominent regulators of host transcriptional immune response, including the APETALA2/ethylene responsive factor (AP2-ERF), the basic Helix-Loop-Helix (bHLH), the NAC (NAM, ATAF1/2, and CUC2), the basic leucine zipper (bZIP) and the WRKY [14]. ERF1 and ORA59 belonging to the AP2/ERF family are notably induced by JA and ET and can be activated synergistically by these two hormones [15, 16]. The MYC2 belonging to the bHLH family has been demonstrated to be an activator of JA response genes (i.e. *VSP2*, *LOX2*), whereas is a negative regulator of JA/ET responsive gene plant defensin 1.2 (*PDF1.2*) that is activated by ERFs [17]. Thus, when the JA response pathway is activated combined with ET, the ERFs branch of the JA response is activated. While the MYC2 activated the independent branch of the JA response without ET [18]. The WRKY family involves modulating numerous host immune responses, particularly WRKY33 [19, 20]. WRKY33 is a central transcriptional regulator of hormone and metabolic responses against *Botrytis cinerea* infection [21]. Recent study links these findings by showing that the ET biosynthetic genes 1-aminocyclopropane-1-carboxylate synthases (*ACS2* and *ACS6*) were induced by GSH in a WRKY33 -dependent manner [22].

Next-generation sequence (NGS) technology based on the Illumina platform is a powerful method for underlying processes of gene expression and secondary metabolism [23]. However, due to the limitations of NGS technology, genes of interest are not completely or accurately assembled leading to unknown errors in analyses [24]. With the development of the sequencing technology, the single molecular real-time (SMRT) sequencing was developed and can overcome these limitations. The SMRT sequencing based on the PacBio platform provides contigs with no gaps and presenting 150-fold to 200-fold improvements and a precise manipulation for subsequent gene cloning work, making it possible to accurately reconstruct full-length splice variants [25]. The technology has been used to characterize the complexity of transcriptomes in *Zea mays* [26], *Sorghum bicolor* [27], and *Populus* [28]. In the development of the stem of *Populus*, the SMRT sequencing complemented Illumina sequencing for quantifying and clarifying transcripts and increasing understanding about dynamic shoot development [28]. Through the integration of the PacBio sequencing and Illumina sequencing, it drastically improved the transcripts of Rice with various alternative splicing (AS), alternative polyadenylation (APA) events, and long non-coding RNAs (lncRNAs) in different developmental stages and growth conditions [29]. Overall, combining NGS and SMRT sequencing can provide high-quality, accurate, and complete isoforms in transcriptome studies, thereby can conducive to the discovery of more AS isoforms, lncRNAs, and fusion genes.

A previous study reported that the canker response mechanism of *M. dometica* was identified using the RNA-seq tool. However, not all the functional transcripts have been identified due to the limitation of NGS. Thus, it is still unclear how the wild apple orchestrates the response to the infection of *V. mali*. Thus, we employed the SMRT sequencing corrected by RNA-seq to generate a full-length transcriptome in wild apple *M. sieversii*. This is the first full-length transcriptome study for the response of wild apple infected with *C. mali*, we obtained 8139 differentially expressed transcripts (DETs) in *M. sieversii* after *V. mali* infection including 544 TFs. These DETs may be related to the transcriptomic dynamics in *M. sieversii* to respond to the infection. Clarification of the process and mechanism of *Valsa* canker disease response in *M. sieversii* can contribution to molecular breeding in which selection of high-quality disease-resistant germplasm through transducing or silencing disease resistance/susceptibility genes.

## Results

### SA and JA contents changes of *M. sieversii* responded to the infection of *V. mali*

**Fig. 1:**
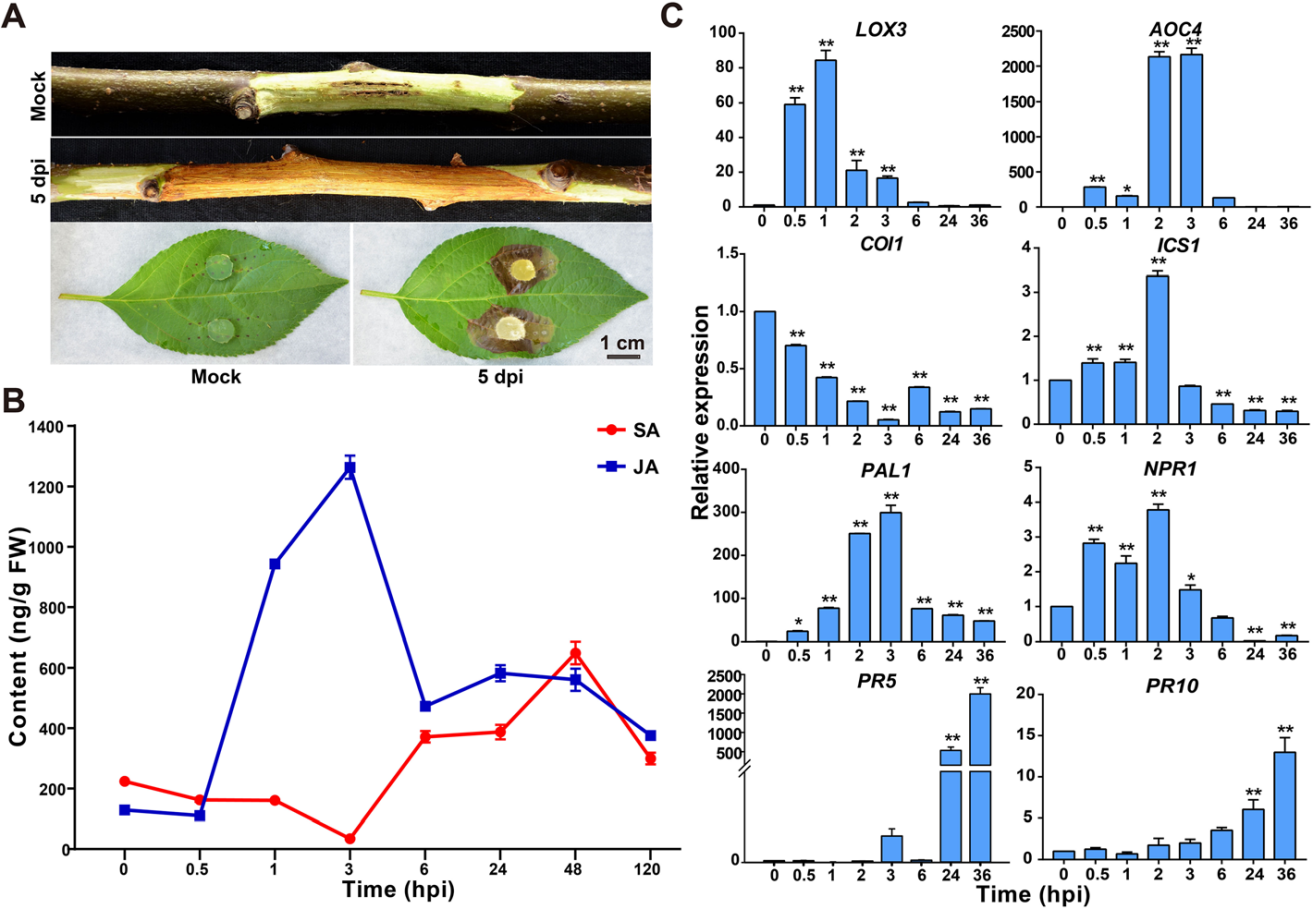
Canker symptoms and SA/JA production changes of *M. sieversii* after *V. mali* infection. **a.** The twigs and leaves of *M. sieversii* inoculated with *V. mali*. Mock: wounds + ddH_2_O, 5 dpi: wounds + *V. mali*; Scale bar, 2 cm. **b.** The productions of free SA and JA (ng/g FW) of twigs inoculated with *V. mali* at 0, 0.5, 1, 3, 6, 24, 48, 120 hpi. **c.** The relative expressions of SA and JA related-genes of twigs inoculated with *V. mali* at 0, 0.5, 1, 2, 3, 6, 24, 36 hpi. *Lipoxygenase 3* (*LOX3*), *allene oxide cyclase 4* (*AOC4*), *coronatine-insensitive protein 1* (*COI1*), *isochorismate synthase 1* (*ICS1*), *phenylalanine ammonia-lyases 1* (*PAL1*), *non-expressor of pathogenesis-related* (*PR*) *genes 1* (*NPR1*), *pathogenesis-related protein 5* (*PR5*), *pathogenesis-related protein 10* (*PR10*). Asterisks indicate significant differences (*p<0.05; **p<0.01; LSD’s test) between each infection timepoints and the 0-hpi control

The necrotic canker symptom in the wounded twig and leaf infected with *V. mali* was observed at 5 dpi (Fig. 1a). To measure the changes of phytohormone levels, we implemented the quantitative hormone measurements of JA and SA at 0, 0.5, 1, 2, 3, 6, 24, 48, 120 h infected with the *V. mali* (Fig. 1b). The production of JA started to increase within 1 h and peaked approximately 10-fold (1262.98 ± 37.76 ng/g FW) at 3 hpi. However, with the increase of the production of SA, the content of JA was reduced accordingly due to antagonistically regulated by the SA from 3 to 6 hpi. Meanwhile, the content of SA was decreased at 3 hpi due to the antagonistic effect of JA. Subsequently, the SA production was increased from 3 to 6 hpi and reached a peak with increased approximately 3-fold (649.10 ± 37.38 ng/g FW) at 48 hpi. From 6 to 120 hpi, the SA and JA presented a consistent pattern such that increased first and then reduced to synergistically respond to the infection. These results imply that the JA-dependent necrotrophic resistance was intensively induced by the invasion of the *V. mali*. A string of signal transductions and transcriptional regulation processes might be triggered after the infection of *V. mali*. Additionally, the relative gene expression of key genes of SA and JA synthesis and signaling transduction pathways were detected by qRT-PCR at 0, 0.5, 1, 2, 3, 6, 24, 36 hpi (Fig. 1c). The relative expression level of *lipoxygenase 3* (*LOX3*) and *allene oxide cyclase 4* (*AOC4*) (JA key synthesis genes) were strongly increased after infection, especially the 80-fold higher expression of *LOX3* at 1 hpi and about 2000-fold expression of *AOC4* from 2 to 3 hpi than 0-hpi control. The gene expression level of *coronatine-insensitive protein 1* (*COI1*) gene, JA signal transduction gene, was slightly reduced after infection. The key SA synthesis genes *isochorismate synthase 1* (*ICS1*) and *phenylalanine ammonia-lyases 1* (*PAL1*) were significantly up-regulated after infection, especially the 300-fold higher expression of *PAL1* at 3 hpi. The expression of *NPR1*, SA key signal transduction gene, was increased from 0.5 to 2 hpi and then decreased after 6 dpi. The *pathogenesis-related protein 5* (*PR5*) and pathogenesis-related protein (*PR10*) were continuously up-regulated after infection with a 2000-fold higher and 13-fold higher increase than control respectively. These results suggested that JA was induced initially to respond to the infection of the necrotrophic pathogen *V. mali*.

### Sequencing of the *M. sieversii* transcriptome infected with *V. mali* using the PacBio platform

**Fig. 2:**
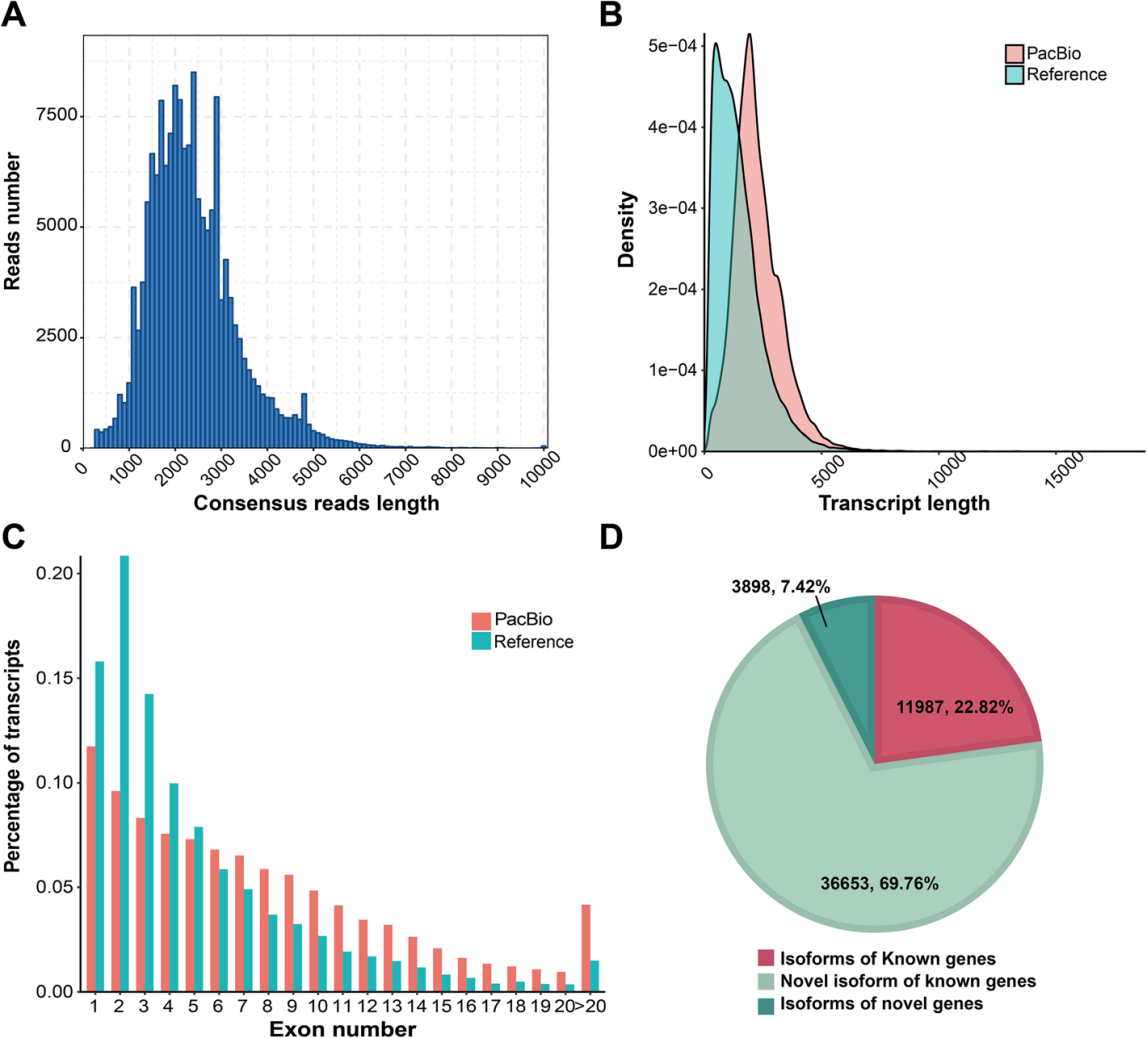
Characterization of *M. sieversii* inoculated with *V. mali* transcripts from PacBio Iso-seq. **a**. The number of consensus reads in different lengths. **b.** Distribution of transcripts lengths. **c.** Distribution of the percentage transcripts with different exon numbers for reference and PacBio Iso-Seq data. **d.** The percentage of PacBio Iso-Seq transcripts that are the known genes, novel transcript of known genes, and transcripts of novel genes

To identify and characterize the transcriptomes of *M. sieversii* twigs inoculated with *V. mali* during different disease response stages, we employed the PacBio SMRT and Illumina sequence technologies for transcriptome. The dynamic transcriptome response to the infection of *V. mali* was examined in twigs of *M. sieversii* at 0, 1, 2, 5 dpi. In the Illumina sequencing data, a total of 164.83 Gb of clean reads were obtained from the twelve samples, and each of these samples contained ≥10.9 Gb of data with Q30 quality scores ≥93.61%. These reads of each sample were mapped uniquely with the ratios from 95.58 to 96% (Additional file 1). The PacBio SMRT sequencing yielded all 12,666,867 subreads (25.71G) with an average read length of 2030 bp, of which 488,689 were full-length non-chimeric reads (FLNC), containing the 5′ primer, 3′ primer and the poly (A) tail (Table 1). The average length of the full-length non-chimeric read was 2264 bp. We used an isoform-level clustering (ICE) algorithm to achieve accurately polished consensuses (Fig. 2a). All these consensuses were corrected using the Illumina clean reads as input data. A total of 159,249 corrected reads were produced using the LoRDEC for the error correction and removal of redundant transcripts, and each represented a unique full-length transcript of average length 2371 bp and N50 of 2596 bp (Table 1). Longer isoforms were identified from Iso-Seq than from the *M. domestica* reference database (GDDH13 v1.0) and more exons were found in this study (Fig. 2b, c). We compared the 52,538 transcripts with the *M. domestica* genome gene set, and they were classified into three groups as follows: (i) 11,987 isoforms of known genes mapped to the *M. domesitica* gene set, (ii) 36,653 novel isoforms of known genes and (iii) 3898 isoforms of novel genes (Fig. 2d). In this study, a high percentage (69.76%) of new isoforms were identified by PacBio full-length sequencing. It suggested that the high percentage of novel isoforms sequenced by SMRT provided a larger number of novel full-length and high-quality transcripts through the correction of RNA-seq.

**Table 1:** Statistics of SMRT sequencing data from samples mixed from 0 to 5 dpi

Sample	Mix0_5d
Subreads base (G)	25.71
Subreads number	12,666,867
Average subreads length (bp)	2030
CCS	633,537
Number of 5′-primer reads	593,825
Number of 3′-primer reads	591,975
Number of Poly-A reads	539,418
Number of FLNC reads	488,689
Average FLNC read length (bp)	2264
FLNC/CCS percentage (FL%)	77.14
Polished consensus reads	159,249
Average consensus reads length (bp)	2362
After correct consensus reads	159,249
After correct average consensus reads length (bp)	2371
N50	2596

### Alternatively spliced (AS) isoform and long non-coding RNA identification

**Fig. 3:**
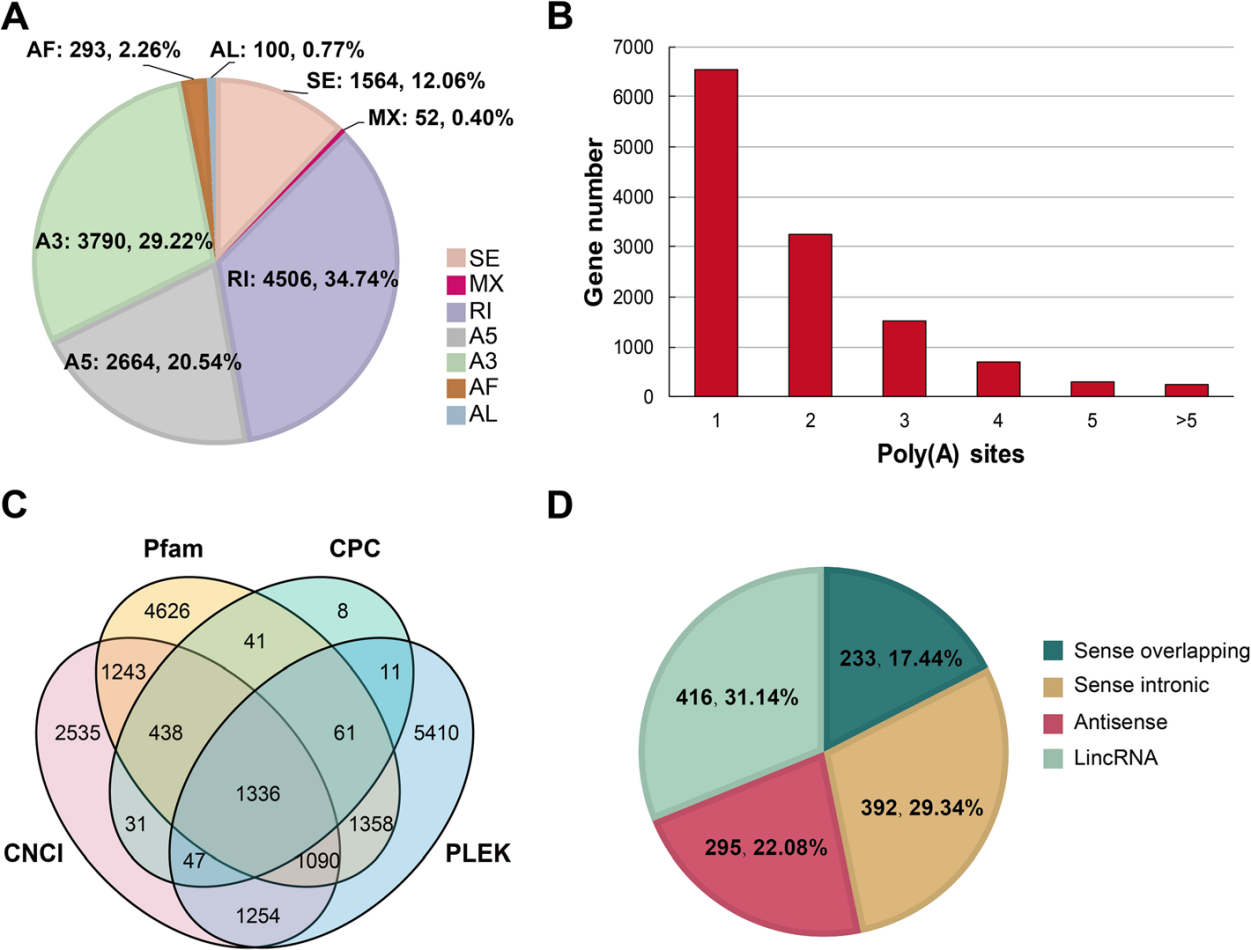
Identification of AS isoforms and lncRNA. **a.** The gene numbers involving AS events. SE: skipped exon; MX: mutually skipped exon; RI: retained intron; A5: alternative 5′ splice site; A3: alternative 3′ splice site; AF: alternative first exon; AL: alternative last exon. **b.** Distribution of the number of poly (A) sites per gene. **c.** Venn diagram of lncRNAs predicted by CNCI, Pfam, CPC, and PLEK methods. **d.** Proportions of four types of lncRNA

**Fig. 4:**
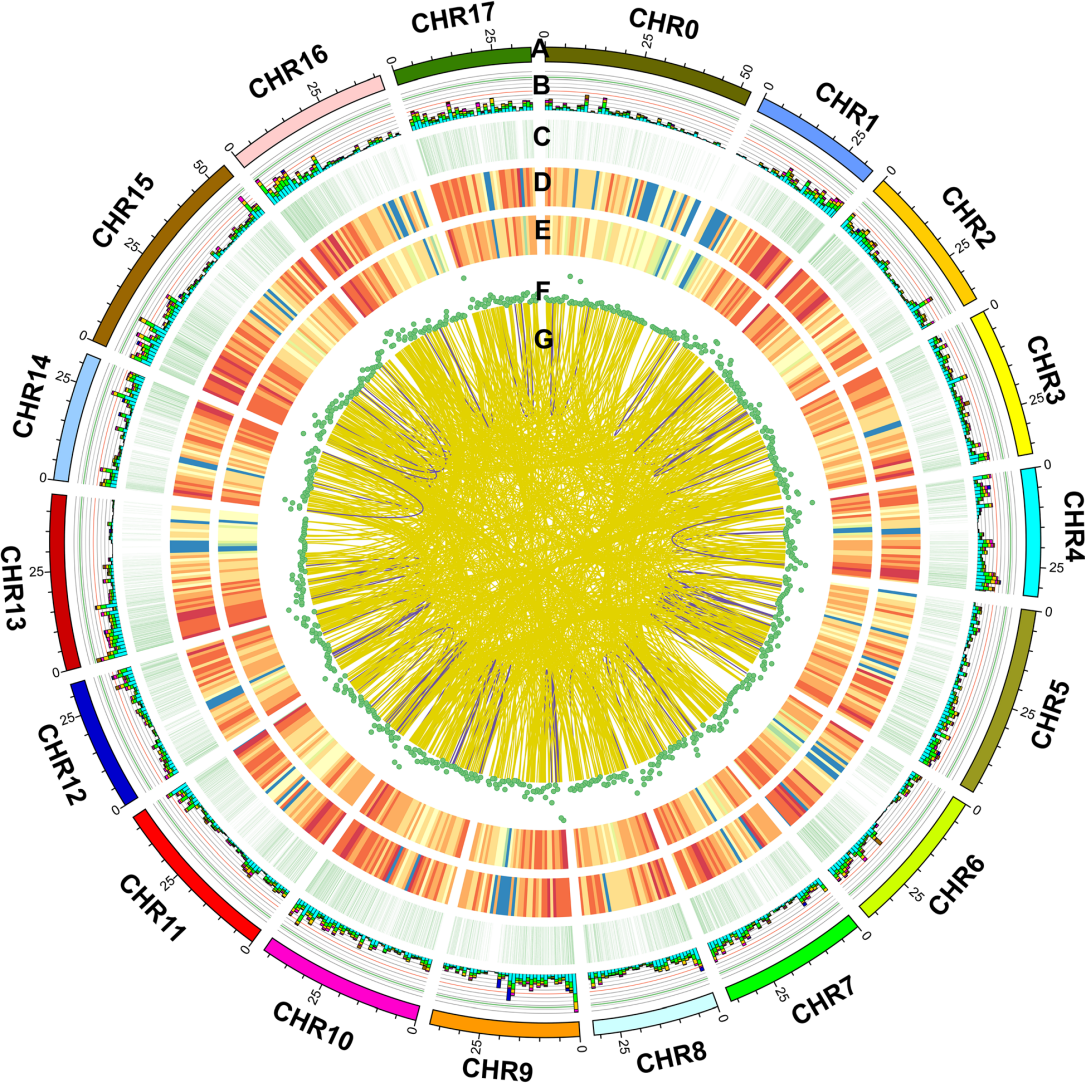
CIRCOS visualization of genomic and transcriptomic features of different response stages (0, 1, 2, and 5 dpi). **a.** Chromosomes of *M. domestica*. **b.** AS position. (Stacking histogram, turquoise: RI; green: A3; yellow: A5; purple: SE; red: MX; brown: AF; dark blue: AL). **c.** APA position mapped to chromosomes. **d.** Novel transcript density. **e.** Novel gene density. **f.** LncRNA density distribution. **g.** Fusion transcript distribution. Intra-chromosome (purple); inter-chromosome (yellow)

AS events in different canker disease response stages were analyzed with SUPPA software. We detected 15,607 genes involved AS events of a total of 20,163 isoforms from the Iso-Seq reads, including skipped exon (SE), mutually exclusive exon (MX), alternative 5′ splice site (A5), alternative 3′ splice site (A3), retained intron (RI), alternative first exon (AF) and alternative last exon (AL). Most AS events in Iso-Seq were RI with several 4506 (Fig. 3a). The exon position was 13,767,261-13,767,364 in chromosome 11 of the reference genome (Additional file 2). To identify accurately differential APA sites in *M. sieversii* during canker disease response, 3′ ends of transcripts from Iso-Seq were investigated. There was a total of 23,737 APA sites of 12,552 genes with at least one APA site (Fig. 3b, Fig. 4, and Additional file 3). We also identified 1602 fusion transcripts (Fig. 4, Additional file 4). Moreover, a total of 1336 lncRNAs were identified by four computational methods from 1168 genes of Iso-Seq. We classified them into 4 groups: 233 sense overlapping (17.44%), 392 sense intronic (29.34%), 295 antisense (22.08%), and 416 lincRNA (31.14%) (Fig. 3c and d). The length of the lncRNA varied from 200 to 6384 bp, with the majority (54.87%) having a length ≤1000 bp, and mapped them to the chromosomes (Fig. 4, Additional file 5). The expression pattern analysis of the lncRNA transcripts based on PacBio transcriptome showed that a total of 277 lncRNA transcripts were significantly differentially expressed in response to the *V. mali* infection (Additional files 5 and 6). GO enrichment analysis of differently expressed lncRNA transcripts showed that in the Molecular Function term, GO-terms “response to toxic substance (GO:0009636)”, “immune response (GO:0006955)”, “response to stimulus (GO:0050896)” and “immune system process (GO:0002376)” were majorly associated with the up-regulated lncRNA transcripts (Additional file 7). It indicated that the differently expressed lncRNA transcripts might play important roles in involving the response to *V. mali* invasion.

### Functional annotations and classifications of DETs

**Fig. 5:**
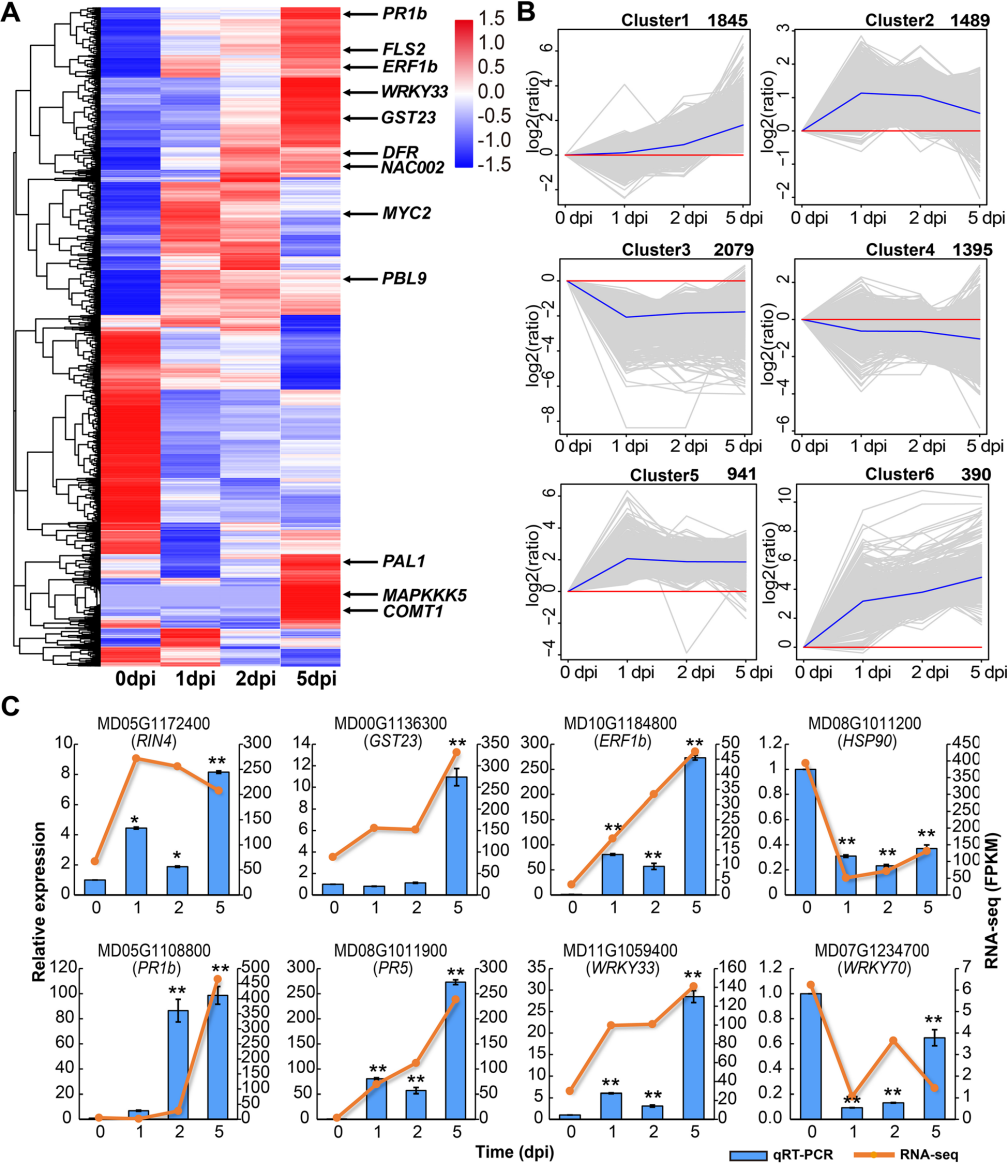
Clustering analysis of the DETs. **a.** Hierarchical clustering graph of 8139 DETs based on the averaged log_10_(FPKM+ 1) values of all genes in each cluster. **b.** 8139 DETs were clustered into six clusters by H-means clustering. The number of genes in each cluster is shown at the top of each cluster. Blue lines show the average values for relative expression levels in each cluster; gray lines represent the relative expression levels of each gene in each cluster; red lines represent the baselines. **c.** Expression pattern for the eight differentially expressed genes during the different disease response stages validated by qRT-PCR. *RPM1-interacting protein 4* (*RIN4*), *glutathione S-transferase 23* (*GST23*), *ET-responsive transcription factor 1b* (*ERF1b*), *heat shock protein 90* (*HSP90*), *pathogenesis-related protein 1b* (*PR1b*), *PR5*, *WRKY transcription factor 33* (*WRKY33*), *WRKY transcription factor 70* (*WRKY70*). The normalized expression level (FPKM) of RNA-seq is indicated on the right y-axis and the relative expression of qRT-PCR is indicated on the right y-axis. qPCR quantitative gene expression data were shown as the mean ± SEM. Asterisks indicate significant differences (*p<0.05; **p<0.01; LSD’s test) between each infection timepoints and the 0-dpi control

To identify key factors involved in the canker disease response stage, we identified 8139 DETs by the PacBio sequencing and 8811 differentially expressed genes (DEGs) based on the RNA-seq data. A total of 2078 DEGs were the overlaps of the Illumina and PacBio transcriptomes. The specificity of the Illumina and PacBio transcriptomes were separately 6733 DEGs and 6061 DETs (Additional file 8). The heatmap of DETs showed that numerous biotic response transcripts were also extensively up and down-regulated in the disease stage (Fig. 5a). Among all these DETs, the most (2079) and the smallest number of DETs (390) were identified as being differentially expressed in the disease response stage. Using the H-means clustering algorithm, 8139 DETs were grouped into 6 clusters (H1-H6) (Fig. 5b). Based on the expression changes in disease response stages (1, 2, and 5 dpi), we identified the disease response related to DETs involved in different response stages.

To determine the functions of resistance (R) genes in *M. sieversii* during the infection of *V. mali*, the expression patterns of DETs involved in the signaling pathway in plant immunity were analyzed. As well known the microbe-associated molecular patterns (MAMPs) recognized genes *Flagellin sensing 2* (*FLS2*) (MD05G1297700) were significantly increased at 5 dpi. The regulator of chitin-induced immunity the *Probable serine/threonine-protein kinase PBL 9* (*PBL9*) (MD07G1199400) and *PBL19* (MD03G1092100 and MD07G1093200) were significantly up-regulated at 5 dpi. Subsequently, the signal transduction related kinase, *mitogen-activated protein kinases* (*MAPKs*), *mitogen-activated protein kinase kinase kinase 5* (*MAPKKK5*) (MD15G1035800) was significantly up-regulated at 5 dpi, which was the consistent expression patterns with *PBLs* (Additional file 9). The expression of *dihydroflavonol 4-reductase* (*DFR*) (MD11G1229100) was significantly increased in the defense-related compound flavonoid biosynthesis process from 2 to 5 dpi. The key gene in lignin formation: *PAL1* (MD12G1116700, MD01G1106900 and MD07G1172700), *caffeic acid 3-O-methyltransferases* (*COMT1*) D01G1089800, MD01G1089900, MD07G1161100, MD07G1209500, MD07G1209600, MD07G1300200, MD07G1300500, and MD12G1103500) which were lignin biosynthesis-related genes, were significantly increased after infection. Besides, the *peroxidase 51* (*PER51*) (MD00G1112500), which can generate the reactive oxygen species (ROS) to respond to the pathogen attack, was continually ascended from 1 to 5 dpi (Fig. 5a, Additional file 9).

To validate the expression pattern of the transcripts in different stages of the canker disease response, we performed qRT-PCR experiments. We selected eight DETs of different aspects of disease response stages with seven DETs showed elevated expression levels and one DET with reduced expression pattern. The qRT-PCR result showed that eight selected DETs have consistent gene expression patterns with RNA-seq data (Fig. 5c). The expression of *ethylene-responsive transcription factor 1b* (*ERF1b*) (MD10G1184800) and *WRKY33* transcription factors (MD11G1059400) were significantly up-regulated from 2 to 5 dpi. The expressions of the plant resistance (R) genes *RPM1-interacting protein 4* (*RNI4*) (MD05G1172400), *pathogenesis-related protein 1b* (*PR1b*) (MD05G1108800)*, PR5* (MD08G1011900), and *glutathione S-transferase 23* (*GST23*) (MD00G1136300) were significantly up-regulated at 5 dpi compared to 0 dpi. Contrarily, expression levels of the *heat shock protein 90* (*HSP90*) (MD08G1011200) and *WRKY70* (MD07G1234700) were significantly down-regulated after infection. This independent qRT-PCR evaluation confirmed the accuracy and reliability of the Illumina sequencing results.

### Different regulatory pathways during the response to the infection of the *V. mali*

Plant defense response to biotic stress involves complex molecular or genetic networks. To further investigate the functions of DETs after the infection of *V. mali*, Gene Ontology (GO) and Kyoto Encyclopedia of Genes and Genomes (KEGG) enrichment were implemented (corrected *P*-value < 0.05).

The results showed that the “UDP-glucosyltransferase activity” (GO: 0035251) was significantly differentially enriched with 37 up-regulated transcripts and 13 down-regulated transcripts at 1 dpi (Additional files 10, and 11). The “oxidoreductase activity” (GO: 0016491) was significantly differentially enriched with 201 up-regulated transcripts and 114 down-regulated transcripts at 2 dpi and with 350 up-regulated transcripts and 92 down-regulated transcripts at 5 dpi, including the *PER51* (Additional files 10, 12, and 13).

**Fig. 6:**
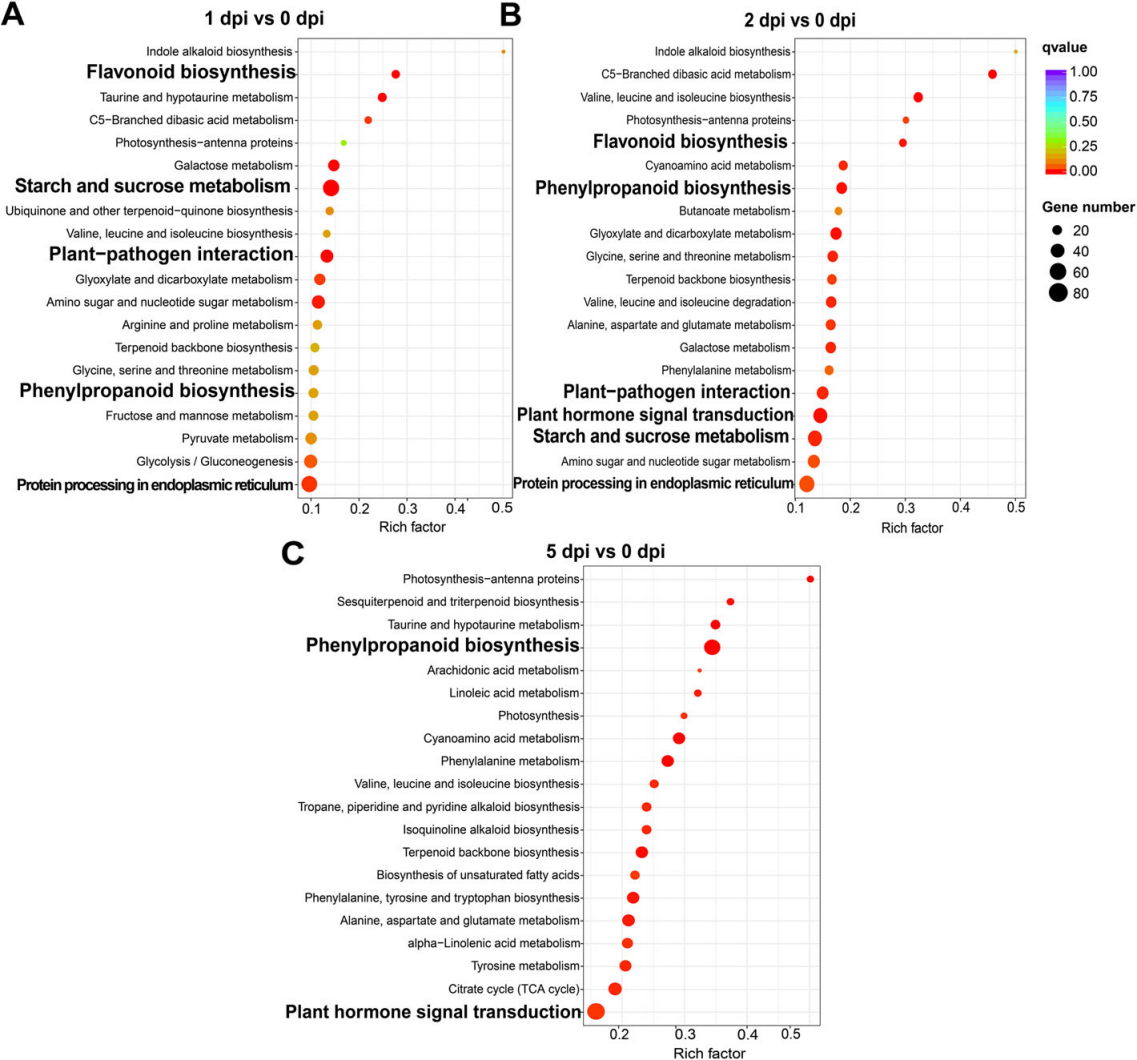
Scatter plot of the enriched TOP 20 KEGG pathways for the DETs during the different disease response stages. **a.** KEGG pathway enrichment on 1 dpi vs 0 dpi. **b.** KEGG pathway enrichment on 2 dpi vs 0 dpi. **c.** KEGG pathway enrichment on 5 dpi vs 0 dpi. A rich factor was the ratio of an input number to the background number in a specific pathway. The size and color of the dots represent the transcript numbers and the q-values, respectively

The enriched TOP 20 KEGG pathways of the DETs were showed based on KEGG enrichment, providing transcripts of genes expression overview during the different canker disease response stages. At the early disease response stage (1 to 2 dpi), four pathways were significantly enriched including “plant-pathogen interaction” (ko04626), “starch and sucrose metabolism” (ko00500), “protein processing in endoplasmic reticulum” (ko04141), and “flavonoid biosynthesis” (ko00941) (Fig. 6a, b, Additional file 14). At the late response stage (5 dpi), the pathway “plant hormone signal transduction” (ko04075) was the most significantly enriched with a rich factor 0.156. Moreover, there were the greatest number of genes (86) in this pathway (Additional file 14). Especially, the pathway “phenylpropanoid biosynthesis” (ko00940) was enriched at both early and late response stages (Fig. 6a-c, Additional file 14). It suggested that these pathways played vital and different roles during the response in *M. sieversii* after the *V. mali* infection.

**Fig. 7:**
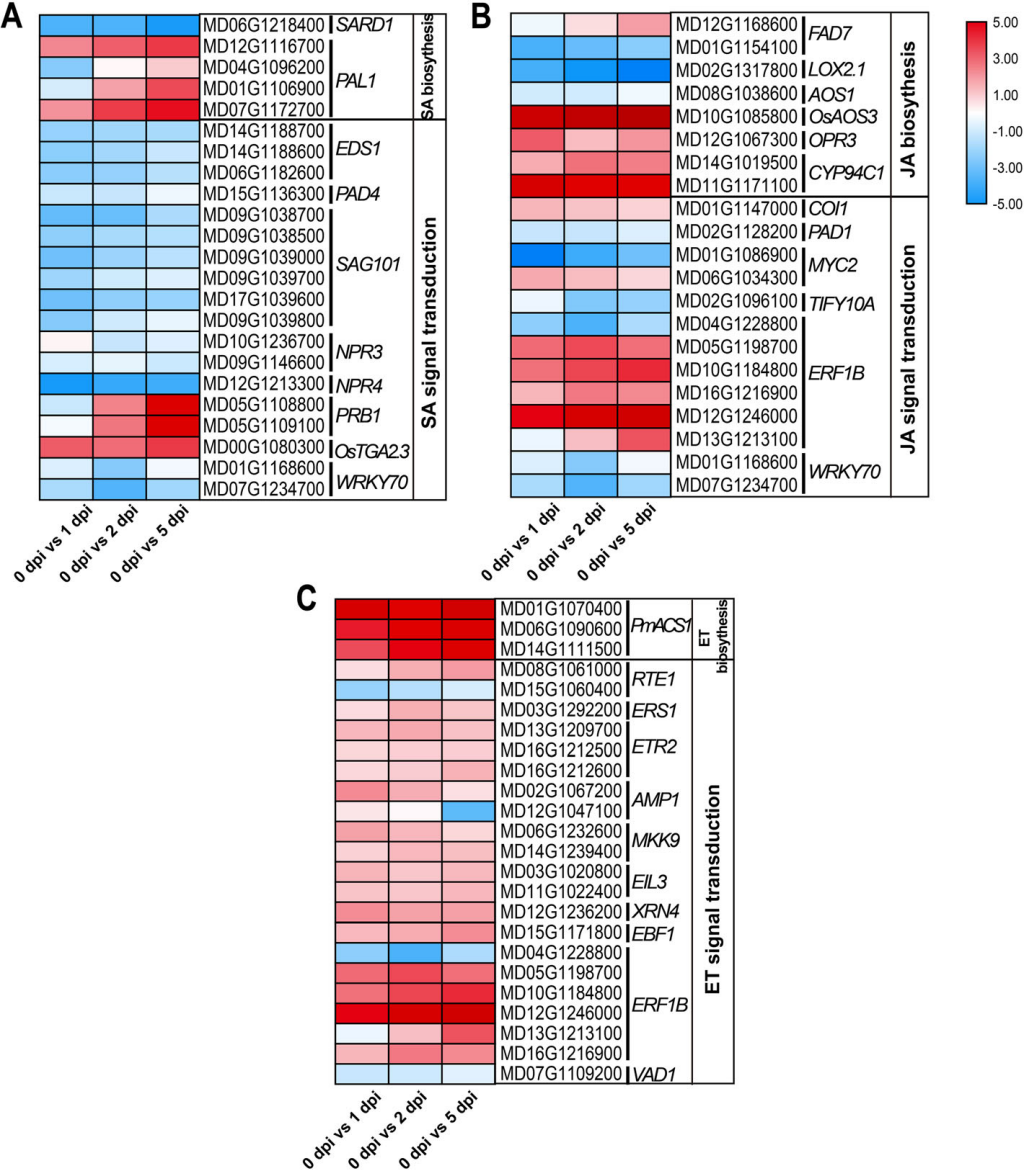
Dynamic changes of phytohormone-related DETs expression during the response to the infection of *V. mali*. **a.** Heatmap of the phytohormone SA pathway. **b.** Heatmap of phytohormone JA pathway. **c.** Heatmap of the phytohormone ET pathway. Heatmaps were generated using log2fold changes of DETs in phytohormone pathways at various stages of disease response

To further study the enrichment pathway “plant hormone signal transduction”, the dynamic changes of phytohormone SA, JA, and ET related DETs expression were presented, during the response to the infection of *V. mali* (Fig. 7). In SA signaling pathway, the necessary key genes *Enhanced Disease Susceptibility1* (*EDS1*) (MD06G1182600, MD14G1188600, and MD14G1188700), *Phytoalexin Deficient4* (*PAD4*) (MD15G1136300) and *Senescence-associated carboxylesterase 101* (*SAG101*) (MD09G1039800, MD17G1039600, MD09G1039700, MD09G1039000, MD09G1038500, and MD09G1038700) in plant systemic acquired resistance (SAR) signal generation and perception were down-regulated from 1 and 5 dpi. However, the marker gene for the SA-mediated signaling pathway *PR1b* (MD05G1109100 and MD05G1108800) was significantly increased (Fig. 7a). JA synthesis-related key genes, *allene oxide synthase 3* (*OsAOS3*) (MD10G1085800), *12-oxo-phytodienoic acid reductases 3* (*OPR3*) (MD12G1067300) and *Cytochrome P450 94C1* (*CYP94C1*) (MD11G1171100 and MD14G1019500) were significantly up-regulated from 1 to 5 dpi. The well-known key player in downstream of JA *COI1* (MD01G1147000) was significantly increased. The *MYC2* (MD06G1034300) core signal transduction mechanism of JA signaling, were significantly up-regulated from 1 to 5 dpi in this pathway. The repressor of JA signaling transduction-related gene *Protein TIFY 10A* (*TIFY10A*) (MD02G1096100) was significantly reduced. The key integrator of JA and ET signals in JA/ET dependent defense, *ERF1b* (MD05G1198700, MD13G1213100, MD10G1184800, MD12G1246000, and MD16G1216900) were significantly increased (Fig. 7b). Meanwhile, ET synthesis-regulated key genes *1-aminocyclopropane-1-carboxylate synthase 1* (*ACS1*) (MD01G1070400, MD06G1090600, and MD14G1111500) were significantly up-regulated from 1 to 5 dpi. The negative regulator *Protein Reversion-to-Ethylene Sensitivity 1* (*RTE1*) (MD15G1060400) was significantly decreased from 1 to 5 dpi. The receptors of ET, *Ethylene response sensor 1* (*ERS1*) (MD03G1292200) and *Ethylene response sensor 1 (ETR2)* (MD13G1209700, MD16G1212500, and MD16G1212600) showed highly increased expression levels at 5 dpi. The ET-responsive transcription factor *ERF1b* (MD05G1198700, MD13G1213100, MD10G1184800, MD12G1246000, and MD16G1216900) were significantly up-regulation from 1 to 5 dpi (Fig. 7c). By combing these dynamic expressions of DETs with the production levels of SA and JA above (Fig. 1c), we determined that the SA and JA antagonistically responded to the infection of *V. mali* at the early response stage, and synergistically. Nevertheless, the dynamic expression of the key signaling genes in the ET pathway showed a similar expression pattern to that in JA. These results imply that JA and ET might regulate the complex response in *M. sieversii* to the infection of *V. mali*.

### Transcription factor dynamics during canker disease response stages

**Fig. 8:**
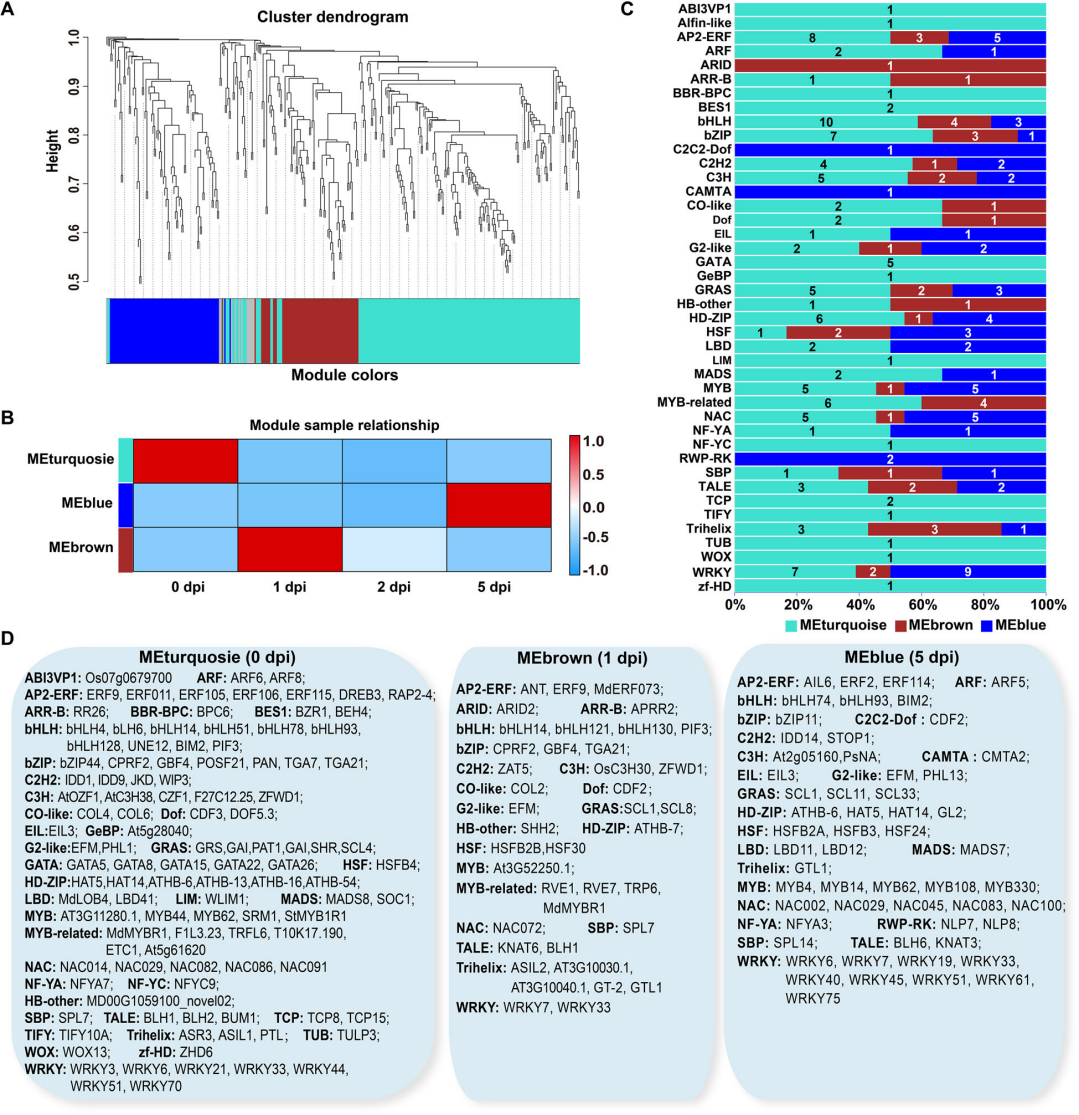
Weighted gene co-expression network analysis (WGCNA) of differentially expressed TFs. **a.** Cluster dendrogram of TFs based on expression levels during disease response stages. Each branch represents a gene and each color below represents a gene co-expression module. **b.** Correlation of transcription factor expression patterns at different time points (0, 1, and 5 dpi). The expression patterns of seven modules are shown by the heatmap. The color bar indicates expression levels from low (blue) to high (red). **c.** Distribution of transcription factor families in different WGCNA modules. MEturquoise: 0dpi; MEbrown: 1dpi; MEblue: 5dpi. Each color represents a co-expression module, and the numbers indicate the number of TFs in the module. **d.** Transcription factor families highly expressed during different disease response stages (0, 1, and 5 dpi)

TFs and transcription regulators (TRs) play important regulatory roles in the plant response process to the *V. mali* infection. Totally 3780 putative TFs, including TFs (2616) and TRs (1164) from 88 families were identified and classified with iTAK. The 523 out of these 2616 TFs were differentially expressed during canker disease response stages. To determine the co-expression and correlation networks of all differentially expressed TFs, weighted correlation network analysis (WGCNA) was conducted (Fig. 8, Additional file 15). Three modules (colored turquoise, brown, and blue) of highly correlated with canker response stage (0, 1, and 5 dpi) were identified (Fig. 8b). Most TFs (140) belong to the turquoise module, in which the peak expression of most TFs was at 0 dpi. The TFs (55) of the brown module and TFs (74) of the blue module have decreased and increased expression from 1 dpi to 5 dpi, respectively (Fig. 8c). Family-specific expression was observed in different stages of the canker disease response (Fig. 8d). Correlation of TOP 4 TFs families peaked at 0 dpi, which were bHLH (10), AP2-ERF (7), bZIP (7) and WRKY (7), including bHLH (*bHLH4*, *bLH6*, *bHLH14*, *bHLH51*, *bHLH78*, *bHLH93*, *bHLH128*, *BIM2*, *PIF3, UNE12*), AP2-ERF (*ERF9*, *ERF011*, *ERF105*, *ERF106*, *ERF115*, *DREB3*, *RAP2*–4), bZIP (*bZIP44*, *CPRF2*, *GBF4*, *POSF21*, *PAN*, *TGA7*, *TGA21*) and WRKY (*WRKY3*, *WRKY6*, *WRKY21*, *WRKY33*, *WRKY44*, *WRKY51*, *WRKY70*). Correlation of TOP 5 TFs families peaked at 1 dpi, including Trihelix (5), bZIP (4), and bHLH (4), MYB_related (4), AP2-ERF (3). Among this, they were that Trihelix (*ASIL2*, *AT3G10030.1*, *AT3G10040.1*, *GT-2*, *GTL1*), bZIP (*CPRF2*, *GBF4*, *TGA21*, *CPRF2*), bHHLH (*bHLH121*, *bHLH130*, *bHLH14*, *PIF3*), MYB_related (*MdMYBR1*, *RVE1*, *RVE7*, *TRP6*), AP2-ERF (*ANT*, *ERF9*, *MdERF073*). At 5 dpi, WRKY (9), MYB (5), NAC (5) AP2-ERF (4), and HD-ZIP (4) were the TOP 5 highly correlated TFs families, which were WRKY (*WRKY6, WRKY7, WRKY19, WRKY33, WRKY40, WRKY45, WRKY51, WRKY61, WRKY75*), MYB (*MYB4*, *MYB14*, *MYB62*, *MYB108*, *MYB330*), NAC (*NAC002, NAC029, NAC045, NAC083, NAC100*), AP2-ERF (*AIL6, ERF2, ERF114, SlPTI5*), and HD-ZIP (*ATHB*-6, *GL2*, *HAT5*, *HAT14*). Among these, the WRKY family was the most abundant type identified in *M. sieversii* in response to *V. mali* infection. These data suggested that TFs in *M. sieversii* were responsible for pathogen stimulus and interaction with other genes during the host response.

## Discussion

### The PacBio sequencing unveils the complexity of the *M. sieversii* infected with *V. mali*

*M. sieversii* is not only an important component of Tianshan Wild Fruit Forest but also an ancestor of *M. domestica* [2]. Previous studies on *M. domestica* canker disease response transcriptome were mainly based on RNA-Seq method [5], which provided that the chitin-receptors responded to the *V. mali* infection and SA/JA signaling were involved in the primary phytohormone pathways. However, the technology limitation of RNA-seq, such as the short reads, false positives, can produce difficulty and inaccuracy to bioinformatics analyses and gene cloning work [30]. SMRT sequencing technology can yield reads of an average length of over 2500–10,000 bp, which can capture large isoform fragments or even full-length isoform transcripts [31]. Although SMRT data has a relatively high error rate, it can provide accurate characterizing of various transcripts corrected with short and high-accuracy Illumina reads [25]. Therefore, combining these two methods, this study provides the first internal transcriptome about the canker disease response of *M. sieversii*. In this work, a total of 164.83 Gb of clean reads were obtained from the transcriptome of twelve samples (0, 1, 2, 5 dpi) contained ≥10.9 Gb of data with Q30 quality scores ≥93.61% by Illumina sequencing. Our SMRT data were of high quality (25.71 G), of which the average full-length reads were enough to catch the full-length transcripts. The PacBio data yield 633,537 circular consensus sequences (CCSs), of which 488,689 were identified as FLNC transcripts. The average length of the FLNC sequences was 2264 bp, which reflected the length of the cDNA sequence in sequencing. A total of 159,249 polished consensuses were identified through an accurate correction with Illumina short reads. We identified 36,653 novel isoforms of know genes (69.76%), 11,987 isoforms of known genes (22.82%), and 3898 isoforms of novel genes (7.43%). The high percentage of novel isoforms of know genes explained that the PacBio full-length sequencing can greatly improve accuracy. The 20,163 AS events, 1336 lncRNAs and 1602 fusion transcripts were also identified. The roles of lncRNAs in various biological processes of plants have been reported that played important roles in flowering [32], reproduction and defense against fungal infections [33]. Five hundred and fourteen lncRNAs of cotton were obtained in the resistance to the *Verticillium dahlia.* Two GhlncNAT-ANX2- and GhlncNAT-RLP7-silenced seedlings displayed an enhanced resistance towards *V. dahliae* and *B. cinerea* through inducing to increase expression levels of the JA positive regulators, *GhLOX1* and *GhLOX2* [34]. In this work, a total of 277 differentially expressed lncRNA transcripts were obtained in response to the *V. mali* infection. The up-regulated lncRNA transcripts were annotated with the GO-terms “response to toxic substance (GO:0009636)”, “immune response (GO:0006955)”, “response to stimulus (GO:0050896)” and “immune system process (GO:0002376)”, which indicates that these lncRNA transcripts play important roles in response to *V. mali* infection. The potential functions of these lncRNAs will need to be further study. By combining the novel transcripts, we improved the *M. sieversii* transcriptome annotation and characterization, providing more comprehensive coverage of gene expression activity and full-length transcripts, it will provide great help for the cloning and utilization of candidate resistance genes in apple.

### The DETs in *M. sieversii* during the response to the *V. mali* infection

Several recent studies reported that AS can generate multiple transcripts from a single gene for contributing to dynamic reprogramming of the plant transcriptome to orchestrate a tightly organized resistance network during plant adaptation to the biotic stress [35]. Splice variants also can increase the functional diversity of proteins [36]. In this study, there were a total of 20,163 isoforms from 15,607 genes found from the PacBio sequencing through mapping with the reference genome. Among this, there were 69.76% novel isoforms of known genes identified. Based on the PacBio and Illumina sequencing data, we identified 8139 DETs and 8811 DEGs in *M. sieversii* during the response to the infection of *V. mali*. We obtained 6061 DETs from PacBio transcriptome, which were different from the Illumina transcriptome. These specific DETs of PacBio transcriptome could be noteworthy in involving in the canker disease response. Compared with the RNA-seq data in *M. domestica* infected with *V. mali* [5], we obtained much more DEGs and DETs. Yet these DETs were absent in transcriptome in *M. domesitca* in the study of Yin et al. In eukaryotes, the APA and AS events are the major processes that contribute to transcriptome diversity [29, 37]. A total of 11,733 isoforms in *Oryza sativa* L. [29], 110,00 non-redundant isoforms in *Zea mays* [26], and 29,730 novel isoforms in *Trifolium pratense* L. [38] were identified using PacBio sequencing. It provides that the SMRT sequencing provides the possibility to obtain full-length sequences and that identify complex splice isoforms, which are difficult to obtain by NGS. Our results could maximize the transcript diversity of wild apple under the infection of necrotrophic pathogen *V. mali* by completing a PacBio full-length sequencing. The identification of the high percentage of novel isoforms of known genes in this study demonstrates that it can provide a comprehensive set of isoforms in *M. sieversii* than Illumina sequencing during the infection of *V. mali*.

Plant cell surface-localized pattern recognition receptors (PRRs) are exploited to sense microbe-derived patterns referred to as pathogen-associated or microbe-associated molecular patterns (PAMPs/MAMPs) [39]. Leucine-rich repeat (LRR) receptor kinases (LRR-RKs), type of PRRs, well-known of AtFLS2 bounds flg22 of bacterial flagellin [40]. And EFR recognizes the conserved N-terminal N-acetylated bacterial elf18 to confer anti-bacterial immunity [41]. The FLS2 and EFR are critical for the priming of pattern-triggered immunity [42]. During the response stage, the *FLS2*, *EFR*, *PBL* were significantly up-regulated. *PBL* is an immediate downstream component of the chitin elicitor receptor kinase 1 (*CERK1*) and contributes to the regulation of chitin-induced immunity in *Arabidopsis* through a MAPK cascade [43, 44]. In this study, the *PBLs* were significantly increased at 5 dpi. Similarly, the *MAPKKK5* was the consistent expression pattern with *PBLs*. It is implied that the *PBL* transcripts (*PBL9*, and *PBL19*) in *M. sieversii* was required for the chitin-induced MAPK activation. Besides, recognition of a pathogen and concomitant signal transduction also can trigger an oxidative burst. The central gene of these reactions, PERs, can catalyze the production of H_2_O_2_, resulting in activation of the plant PCD [45]. In this study, *PER51* was continually increased from 1 to 5 dpi, implied that the *M. sieversii* host applied the oxidative burst to activate the PCD to inhibit the *V. mali* invasion. However, excessive ROS could be toxic to plant cells. To offset potentially negative effects of ROS, the GSTs can help balance the HR response avoiding causing damage to plants [46]. The *GST23* in *M. sisversii* was significantly high expression at 5 dpi. It was inferred that the *GST23* could be closely related with detoxication.

Based on the GO and KEGG enrichment analysis of DETs, we identified several genes during the canker disease response stages in *M. sieversii*. Well-known ROS is produced via the enhanced enzymatic activity of cell wall-bound peroxidases in plant pathogen defense for activation of the programmed cell death (PCD) [45]. In this study, the “UDP-glucosyltransferase activity” and oxidoreductase activity” were significantly differentially enriched during the response. The “starch and sucrose metabolism” pathway was significantly enriched at the early stage response to *V. mali* infection. It may contribute to cell wall synthesis and lesion repair, which is consistent with the previous study [47]. The “protein processing in endoplasmic reticulum” enriched pathway in this study, could involve in the immune response to the *V. mali*. According to the previous report, this pathway may contribute to the plant resistance mechanism [48]. Based on the KEGG analysis, the “plant hormone signal transduction” pathway was enriched, including JA, SA, ET, and other phytohormones. It was consistent with the RNA-seq data in *M. domestica* from Yin et al. (2016). As the SA/JA hormone level measurements in our study proved that JA and SA were exactly involved in the response to the *V. mali* infection. Phenylpropanoid biosynthesis is central to secondary metabolite production of defense-related compounds including flavonoid and lignin [49, 50]. In cotton plants, lignin improved the resistance to defense response to *Verticillium dahlia* infection [51]. In this study, the phenylpropanoid biosynthetic genes were mostly activated from 2 to 5 dpi, which the comprised transcripts are key genes in lignin formation: *PAL1, COMT1*. It is consistent with the RNA-seq analysis in *M. domesitca* by Yin et al. (2016). The key transcript of *DFR* was significantly differentially changed in the flavonoid biosynthesis process in response to infection. Additionally, ROS can not only involve in HR to produce cell death to defend the invasion of the canker fungal but also lead to physical reinforcement of the plant cell wall. In our data, the ROS generated gene *PER51* was continually ascended from 1 to 5 dpi. Overall, the functional and numerical changes in DETs reflected the highly dynamic and organized changes in gene expression responses of *M. sieversii* to respond to the infection of *V. mali*.

### JA, ET, and SA modulate the response in *M. sieversii* to the *V. mali* infection

Phytohormones SA, JA, and ET play an important role in the regulation of different signaling pathways in plant defense to distinct pathogens [52]. JA plays an important role in defense response against necrotrophic pathogens and herbivores [10, 53, 54]. We determined that the JA production was initially produced to respond to the necrotrophic pathogen *V. mali* infection from 0.5 to 3 hpi and antagonistically inhibited with the increased SA production. However, with the increase of SA production, the JA production was drastically reduced at 6 hpi. It was consistent with the classic antagonism between SA and JA [7]. Subsequently, both the SA and JA level presented consistency after 24 hpi based on the reduction of the JA production, which increased at 2 dpi and decreased at 5 dpi. It may show a transient synergistic enhancement when the SA and JA were at relatively low concentrations [55]. According to the kinetics of SA-dependent suppression of JA signaling, the suppression of SA was completely absent when the SA was applied more than 30 h [56]. Additionally, we proved that JA/SA-related genes (*LOX3*, *AOC4*, *COI1*, *PAL1*, *ICS1*, *NPR1*) played important roles at the transcription level using the FPKM values from RNA-seq and relative transcript abundance from qRT-PCR in response to infection. Furthermore, activation of JA can get synergistically transduced with the ET response [10]. We determined that the ET-synthesis related gene *ACS1* was significantly continuously increased. Besides the expressions of the ET receptor (*ERS1* and *ETR2*) showed highly increased levels after infection. We speculated that the ET could be actively involved in the defense response to the infection of *V. mali*. Additionally, the expression pattern of ET-related key genes could represent a consistent expression pattern with which of JA. It inferred that JA and ET could operate synergistically in regulating the defense-related genes to respond to the *V. mali* infection.

### Differentially expressed TFs response to the *V. mali* infection

Plant TFs are central players that interacted with other co-regulators to establish transcription regulatory networks to orchestrate host immunity [14]. Major plant TF families, including AP2-ERF, bHLH, NAC, TGA/bZIP, and WRKY involved in response to biotic stresses [57]. In this study, the members of the Trihelix, bZIP, bHLH, MYB_related, and AP2-ERF families were involved in the response to the early stage the invasion of *V. mali* (1 dpi), then the members of WRKY, MYB, NAC, AP2-ERF, and HD-ZIP families contributed to the defense at the late stage (5 dpi) for *V. mali* infection. The ERF subfamily members are reported to involve in the regulation of genes responsive to biotic stress, in particular to genes related to the JA and ethylene hormone signaling pathways [57]. In *Arabidopsis*, the ERF2 can be induced by MeJA for enhanced resistance against the fungal pathogen, and then activates pathogen-responsive genes *PDF1.2*, *Th2.1* and *PR4* (basic chitinase) [58]. In our data, *ERF2* was significantly differentially raised at the late stage response, indicating that *ERF2* could be involved in the plant immune response in *M. sieversii* to *V. mali* infection. The WRKY family are major players in coping with various biotic stresses [59, 60]. *AtWRKY33* is critical for mediating immune resistance toward the necrotrophic fungus *B. cinerea* via negative regulation of ABA signaling [19]. AtWRKY33 also can induce the expression of the JA-regulated *PDF1.2* gene to enhances resistance to the *B. cinerea* [61]. In rice, OsWRKY45 improves the resistance toward both bacterial and fungal pathogens, whereby the two alleles OsWRKY45–1 and OsWRKY45–2, play opposite roles in the partial resistance toward the bacterium *Xoo* [60]. *AtWRKY70* integrates signals for antagonistic pathways through activating SA-induced genes and repressing JA-responsive genes [12]. In this study, *WRKY33* was abundant in RNA-seq data and detected by qRT-PCR from 1 to 5 dpi. Combining analysis with the JA and SA level from 1 to 5 dpi, we inferred that *WRKY33* played an important role in regulating the JA signaling transduction in *M. sieversii* to response to the infection of *C. mali*. Additionally, the *WRKY6, WRKY7, WRKY19, WRKY33, WRKY40, WRKY45, WRKY51, WRKY61, WRKY75* were significantly differential expressions at 5 dpi (Fig. 8d). These WRKY and AP2-ERF TFs may involve in the JA/ET-induced defense, but the potential functions will need to be experimentally verified.

## Conclusions

In conclusion, we determined that JA responds positively to the necrotrophic pathogen *V. mali* invasion. SA antagonistically inhibits the JA hormone level at the early response stage and then synergistically in regulating the late response stage. We manipulated the PacBio full-length transcriptome analysis to elaborate on the underlying mechanism of the response in wild apple. The phytohormone signal pathway regulatory played an important role in the response stage. Additionally, the enriched disease resistance pathways and differentially expressed TFs dynamics collectively contributed to the immune response. The long-read PacBio sequencing analysis unveils the dynamic complexity of the *M. sieversii* transcriptome after *V. mali* infection, it will promote the molecular mechanism revealing of apple response to the *Valsa* canker disease, and provided potential gene resources for further anti-pathogen molecular breeding.

## Methods

### Sample collection and pathogen infection

Twigs of *M. sieversii* were collected in May 2017 from the area (43**°** 23′ 2.20′′ N; 83**°** 35′43.48′′ E) in a natural Wild Reserve Forest in Yili, Xinjiang. These samples were allowed to be obtained from the wild with permission from the Forest Bureau of Xinyuan County. The germplasm of *M. sieversii* was identified by Ph.D. Wenjun Li, who worked in Xinjiang Institute of Ecology and Geography, Chinese Academy of Sciences. The twigs amputated from the identical tree were surface sterilized and inoculated with minor modifications as described by Wang et al. [62]. Healthy twig segments (15 mm in diameter) of one tree were washed with ddH_2_O, immersed in 70% ethanol for 10 min, and then rinsed with ddH_2_O. These sterilized twigs were punctured with a fabric pattern wheel (2 cm in diameter) and inoculated with a mycelial plug (5 mm) excised aseptically from the edge of a 5-day-old canker pathogen *V. mali* (EGI1) on PDA media [4]. All inoculated twigs were incubated at 25 °C in darkness and under high humidity (90%RH) for 5 days. Barks of twigs near the canker were separately harvested at the time points of 0, 1, 2, and 5 dpi and each sample contained three biological replicates. Bark samples from 0 dpi time point were collected for RNA extraction as controls. All samples were immediately frozen in liquid nitrogen after collection and stored at − 80 °C for follow-up experiments. The Illumina sequencing was conducted using twelve samples (0, 1, 2, 5 dpi) and the PacBio sequencing was implemented using the mixture of the samples.

### Phytohormone analysis

Plant hormones of free SA and JA productions were extracted according to a previously described method [63, 64]. SA and JA were extracted and quantified according to the method of Liu et al. with appropriate modifications [65]. Briefly, twig samples (0.5 g for each sample) were immediately frozen in liquid nitrogen and ground with pestle and mortar. The ground samples were extracted with 500 μL modified Bieleski solvent (methanol/H_2_O, 80/20, v/v) at 4 °C for 12 h. The solutions of SA and JA were prepared as internal standards at a concentration of 1 μg/mL in 100% methanol. All nano-LC experiments were performed on a Shimadzu Prominence nano-flow liquid chromatography system (Kyoto, Japan) with two LC-20 AD nano pumps, two vacuum degassers, a LC-20AB HPLC pump, a SIL-20 AC HT autosampler, and a FCV nano valve. The analytical column of poly (MAA-co-EDMA) monolithic column (100 μm i.d., 360 μm o.d., 30-cm long, purchased from Weltech Co., Ltd., Wuhan, China) was connected to the nano-LC system and conditioned with the mobile phase (ACN/H_2_O, 50/50, v/v) at a flow rate of 600 μL/min for 30 min.

### RNA quantification and qualification

Total RNA of each biological sample was isolated using a Spectrum Plant Total RNA Kit (Sigma-Aldrich, USA). RNA concentration was measured by Qubit RNA Assay Kit in Qubit 2.0 Fluorometer (Life Technologies, CA, USA). RNA integrity was assessed by the RNA Nano 6000 Assay Kit of the Bioanalyzer 2100 system (Agilent Technologies, CA, USA).

### Illumina RNA-seq library construction and sequencing

A total of 3 μg RNA per sample was used as input material for the RNA sample preparations. Sequencing libraries were generated using NEBNext® Ultra™ RNA Library Prep Kit for Illumina (NEB, USA) following the manufacturer’s recommendations and index codes were added to attribute sequences to each sample. The library preparations were sequenced on an Illumina Hiseq platform and 125 bp–150 bp paired-end reads were generated. HTSeq v0.6.1 was used to count the reads numbers mapped to each gene. And then FPKM of each gene was calculated based on the length of the gene and reads count mapped to this gene. Differential expression analysis of three biological replicates per condition was performed using the DESeq R package (1.18.0). The resulting *P*-values were adjusted using Benjamini and Hochberg’s approach for controlling the false discovery rate. Transcripts with an adjusted *P*-value < 0.05 and log_2_ fold change ≥ 2 found by DESeq were assigned as differentially expressed. Clustering patterns of DETs during different response stages were determined by cluster analysis of all DETs using the Euclidean distance method associated with complete linkage [66, 67].

### Library preparation and SMRT sequencing

The Iso-Seq library was prepared according to the Isoform Sequencing protocol (Iso-Seq) using the Clontech SMARTer PCR cDNA Synthesis Kit and the BluePippin Size Selection System protocol as described by Pacific Biosciences (PN 100–092–800-03). Sequence data were processed using the SMRTlink 5.0 software. The CCSs from subread BAM files (parameters: min length = 200, max drop fraction = 0.8, no polish TRUE, min z-score = − 9999, min passes = 1, min predicted accuracy = 0.8, max length = 18,000) were classified into full length and non-full length reads using pbclassify.py script, ignore poly-A false, min Seq Length 200. Non-full length and full-length reads were then got into the clustering step, which does isoform-level clustering (ICE), followed by final arrow polishing. Additional nucleotide errors in consensus reads were corrected using the Illumina RNAseq data with the software LoRDEC [68].

### Mapping to the reference genome and gene structure analysis

Reference genome and gene model annotation files were downloaded from the genome website directly (https://iris.angers.inra.fr/gddh13/the-apple-genome-downloads.html). Aligning consensus reads to reference using the genome mapping and alignment program (GMAP, version 2017-01-14) [69]. Gene structure analysis was performed by the TAPIS pipeline (version 1.2.1, https://bitbucket.org/comp_bio/tapis) [27]. The GMAP output bam format file and gff/gtf format genome annotation file were used for gene and transcript determination. Alternative splicing events were identified using the SUPPA (version: 2017-02-07, https://bitbucket.org/regulatorygenomicsupf/suppa) [70]. It generates different alternative splicing event types: SE, MX, A5, A3, RI, AF and AL. APA events were then analyzed by TAPIS described previously [70]. Fusion transcripts were determined as transcripts mapping to two or more long-distance range genes and were validated by at least two Illumina reads [26]

### LncRNA identification

Transcripts were predicted using four computational approaches, including coding-non-coding index (CNCI) [71], coding potential calculator (CPC) [72], a predictor of lncRNAs and messenger RNAs via an improved k-mer scheme (PLEK) [73], and Pfam database [74] to identify lncRNA candidates. The lncRNAs were divided into four groups: sense overlapping, sense intronic, antisense, and lincRNA based on the method reported by Harrow [75].

### TF identification and analysis

Transcription factors were predicted using iTAK software and assign genes to different families [76]. The WGCNA package (v1.42) was used to construct co-expression networks [77]. Transcripts of TFs with FPKM values > 1 were used for WGCNA co-expressed network analysis. The modules were obtained using the automatic network construction function blockwiseModules with default settings.

### Transcripts functional annotation

Corrected transcripts were annotated based on the following databases: NR (NCBI non-redundant protein sequences), NT (NCBI non-redundant nucleotide sequences), Pfam (http://pfam.sanger.ac.uk/), KOG/COG (http://www.ncbi.nlm.nih.gov/COG/), Swiss-Prot (http://www.expasy.org/sprot/), KEGG Ortholog database (http://www.genome.jp/kegg), GO (http://www.geneontology.org). We used the software of BLAST (version 2.2.26) and set the e-value ‘1e-10’ in NT database analysis. GO annotations were determined based on the Diamond BLASTX software and set the e-value ‘1e-10’ in NR, KOG, Swiss-Prot, and KEGG database analysis. The functional categorization of DETs was performed using MapMan 3.6.0RC1 [78].

### Validation of DETs by qRT-PCR

The qRT-PCR assays were conducted to validate the consistency of RNA-Seq analysis. Five-microgram total RNA was eliminated genomic DNA. The cDNA was synthesized using the PrimeScript RT reagent Kit with gDNA Eraser (Takara, Dalian, China). Primers of the disease resistance-related DEGs sequences (Additional file 16) were designed using Primer-BLAST (https://www.ncbi.nlm.nih.gov/tools/primer-blast/). The expression of the *EF1a* gene was used as an internal control [79]. Quantitative reverse transcription PCR was carried out with the TB Green™ Premix Ex Taq™ II (Tli RNaseH Plus) (Takara) on a CFX96 Real-Time PCR Detection System (Bio-Rad, USA). Relative gene expression levels were calculated using the 2^-ΔΔCt^ method [80].

## Supplementary Information

**Additional file 1. **Statistics of Illumina RNA sequencing data from twigs in *M. sieversii* inoculated with the *V. mali* at 0, 1, 2 and 5 dpi.

**Additional file 2.** Details regarding AS.

**Additional file 3.** Details regarding APA.

**Additional file 4.** Details regarding fusion gene.

**Additional file 5.** Details regarding lncRNA.

**Additional file 6.** The expression patterns and H-clusterings of the differentially expressed lncRNAs.

**Additional file 7.** GO-term enrichments of the differentially expressed lncRNAs.

**Additional file 8.** Lists of DETs and DEGs.

**Additional file 9.** Details regarding DETs.

**Additional file 10.** Details regarding enriched GO term of DETs.

**Additional file 11: **Directed acyclic graph (DAG) visualization of enriched GO terms for DETs of *M. sieversii* in response to the *V. mali* infection at 1 dpi vs 0 dpi.

**Additional file 12: **DAG visualization of enriched GO terms for DETs of *M. sieversii* in response to the *V. mali* infection at 2 dpi vs 0 dpi.

**Additional file 13: **DAG visualization of enriched GO terms for DETs of *M. sieversii* in response to the *V. mali* infection at 5 dpi vs 0 dpi.

**Additional file 14.** Details regarding enriched KEGG pathway of DETs.

**Additional file 15.** Details regarding differentially expressed TFs of each modules in WGCNA analysis.

**Additional file 16.** Details regarding the qRT-PCR primers.

## Data Availability

The datasets generated during the current article are available at the Sequence Read Archive (SRA) database of National Center for Biotechnology Information (NCBI: https://www.ncbi.nlm.nih.gov/) under project accession number PRJNA687214 (https://dataview.ncbi.nlm.nih.gov/object/PRJNA687214?reviewer=dve4c2uosci45ag7dnbunbrt44) and the additional files. Reference genome of GDDH13 Version 1.1 and gene model annotation files are available at the genome website directly (https://iris.angers.inra.fr/gddh13/the-apple-genome-downloads.html).

## Abbreviations

JA: Jasmonic acid
SA: Salicylic acid
PacBio: Pacific biosciences
DETs: Differentially expressed transcripts
APA: Alternative polyadenylation
TFs: Transcription factors
ET: Ethylene
*NPR1*: Non-expressor of pathogenesis-related (PR) genes1
AP2-ERF: APETALA2/ethylene responsive factor
bHLH: Basic helix-loop-helix
NAC: NAM, ATAF1/2, and CUC2
bZIP: Basic leucine zipper
*PDF1.2*: Plant defensin 1.2
NGS: Next-generation sequence
SMRT: Single molecular real-time
AS: Alternative splicing
lncRNAs: Long non-coding RNAs
*LOX3*: Lipoxygenase 3
*AOC4*: Allene oxide cyclase 4
*COI1*: Coronatine-insensitive protein 1
*ICS1*: Isochorismate synthase 1
*PAL1*: Phenylalanine ammonia-lyases 1
*PR5*: Pathogenesis-related protein 5
*PR10*: Pathogenesis-related protein
FLNC: Full-length non-chimeric reads
ICE: Isoform-level clustering
CCSs: Circular consensus sequences
SE: Skipped exon
MX: Mutually skipped exon
RI: Retained intron
A5: Alternative 5′ splice site
A3: Alternative 3′ splice site
AF: Alternative first exon
AL: Alternative last exon
DEGs: Differentially expressed genes
MAMPs: Microbe-associated molecular patterns
*FLS2*: Flagellin sensing 2
*PBL9*: Probable serine/threonine-protein kinase PBL 9
MAPKs: Mitogen-activated protein kinases
*MAPKKK5*: Mitogen-activated protein kinase kinase kinase 5
*DFR*: Dihydroflavonol 4-reductase
*COMT1*: Caffeic acid 3-O-methyltransferase
*PER51*: Peroxidase 51
ROS: Reactive oxygen species
*ERF1b*: Ethylene-responsive transcription factor 1b
*RNI4*: RPM1-interacting protein 4
*GST23*: Glutathione S-transferase 23
*HSP90*: Heat shock protein 90
GO: Gene Ontology
KEGG: Kyoto encyclopedia of genes and genomes
*EDS1*: Enhanced disease susceptibility1
*PAD4*: Phytoalexin deficient4
*SAG101*: Senescence-associated carboxylesterase 101
SAR: Systemic acquired resistance.
*AOS3*: Allene oxide synthase 3
*OPR3*: 12-oxo-phytodienoic acid reductases 3
*CYP94C1*: Cytochrome P450 94C1
*TIFY10A*: Protein TIFY 10A
*ACS1*: 1-Aminocyclopropane-1-carboxylate synthase 1
*RTE1*: Protein reversion-to-ethylene sensitivity 1
*ERS1*: Ethylene response sensor 1
*ETR2*: Ethylene response sensor 1
TRs: Transcription regulators
WGCNA: Weighted correlation network analysis
PRRs: Pattern recognition receptors
PAMPs/MAMPs: Pathogen-associated or microbe-associated molecular patterns
LRR-RKs: Leucine-rich repeat (LRR) receptor kinases
*CERK1*: Chitin elicitor receptor kinase 1
PCD: Programmed cell death
