# Supplementary material for: PacBio full-length transcriptome of wild apple (Malus sieversii) provides insights into canker disease dynamic response
Source: BMC Genomics. 2021 Jan 14;22:52. doi: 10.1186/s12864-021-07366-y (PMC7809858; doi:10.1186/s12864-021-07366-y)
Supplement: Supplementary file 1 — Additional file 1. Statistics of Illumina RNA sequencing data from twigs in M. sieversii inoculated with the V. mali at 0, 1, 2 and 5 dpi. [file 12864_2021_7366_MOESM1_ESM.docx]

**Table S1** Statistics of Illumina RNA sequencing data from twigs in *M. sieversii* inoculated with the *C. mali* at 0, 1, 2, 5 dpi.

| Sample name | Raw reads | Clean reads | Clean bases (G) | Error rate (%) | Q30 (%) | GC content (%) | Total mapped |
| --- | --- | --- | --- | --- | --- | --- | --- |
| WW_0d_1 | 100054628 | 100054628 | 15.01 | 0.03 | 93.88 | 46.91 | 95857695 (95.81%) |
| WW_0d_2 | 95586376 | 95586376 | 14.34 | 0.03 | 94.06 | 47.11 | 91528209 (95.75%) |
| WW_0d_3 | 104582186 | 104582186 | 15.69 | 0.03 | 93.89 | 47.07 | 100111085 (95.72%) |
| WF_1d_1 | 103943346 | 103943346 | 15.59 | 0.03 | 94.01 | 47.28 | 99548150 (95.77%) |
| WF_1d_2 | 82216182 | 82216182 | 12.33 | 0.03 | 93.88 | 47.48 | 78583178 (95.58%) |
| WF_1d_3 | 76739052 | 76739052 | 11.51 | 0.03 | 93.88 | 47.51 | 73567532 (95.87%) |
| WF_2d_1 | 84643736 | 84643736 | 12.7 | 0.03 | 93.82 | 47.2 | 81245905 (95.99%) |
| WF_2d_2 | 102031252 | 102031252 | 15.3 | 0.02 | 94.19 | 47.25 | 97750875 (95.8%) |
| WF_2d_3 | 94236976 | 94236976 | 14.14 | 0.03 | 93.61 | 47.04 | 90305942 (95.83%) |
| WF_5d_1 | 92526112 | 92526112 | 13.88 | 0.03 | 94.02 | 46.93 | 88829356 (96%) |
| WF_5d_2 | 72095162 | 72095162 | 10.81 | 0.03 | 93.83 | 46.98 | 69162398 (95.93%) |
| WF_5d_3 | 90205540 | 90205540 | 13.53 | 0.03 | 93.74 | 47.12 | 86484141 (95.87%) |
