# Supplementary figures and images for: PacBio full-length transcriptome of wild apple (Malus sieversii) provides insights into canker disease dynamic response
Source: BMC Genomics. 2021 Jan 14;22:52. doi: 10.1186/s12864-021-07366-y (PMC7809858; doi:10.1186/s12864-021-07366-y)

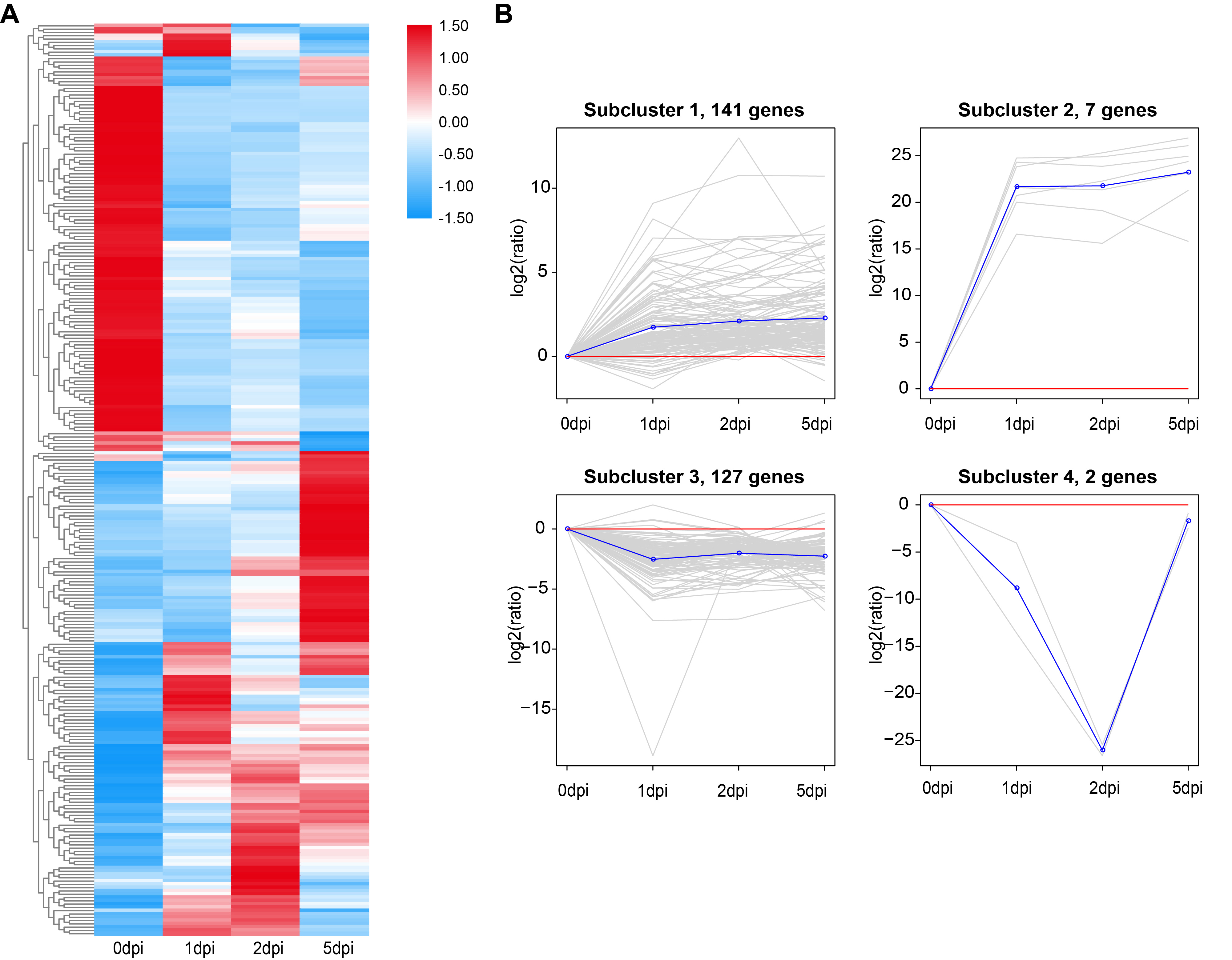

Supplement: Supplementary file 6 — Additional file 6. The expression patterns and H-clusterings of the differentially expressed lncRNAs. [file 12864_2021_7366_MOESM6_ESM.jpg]

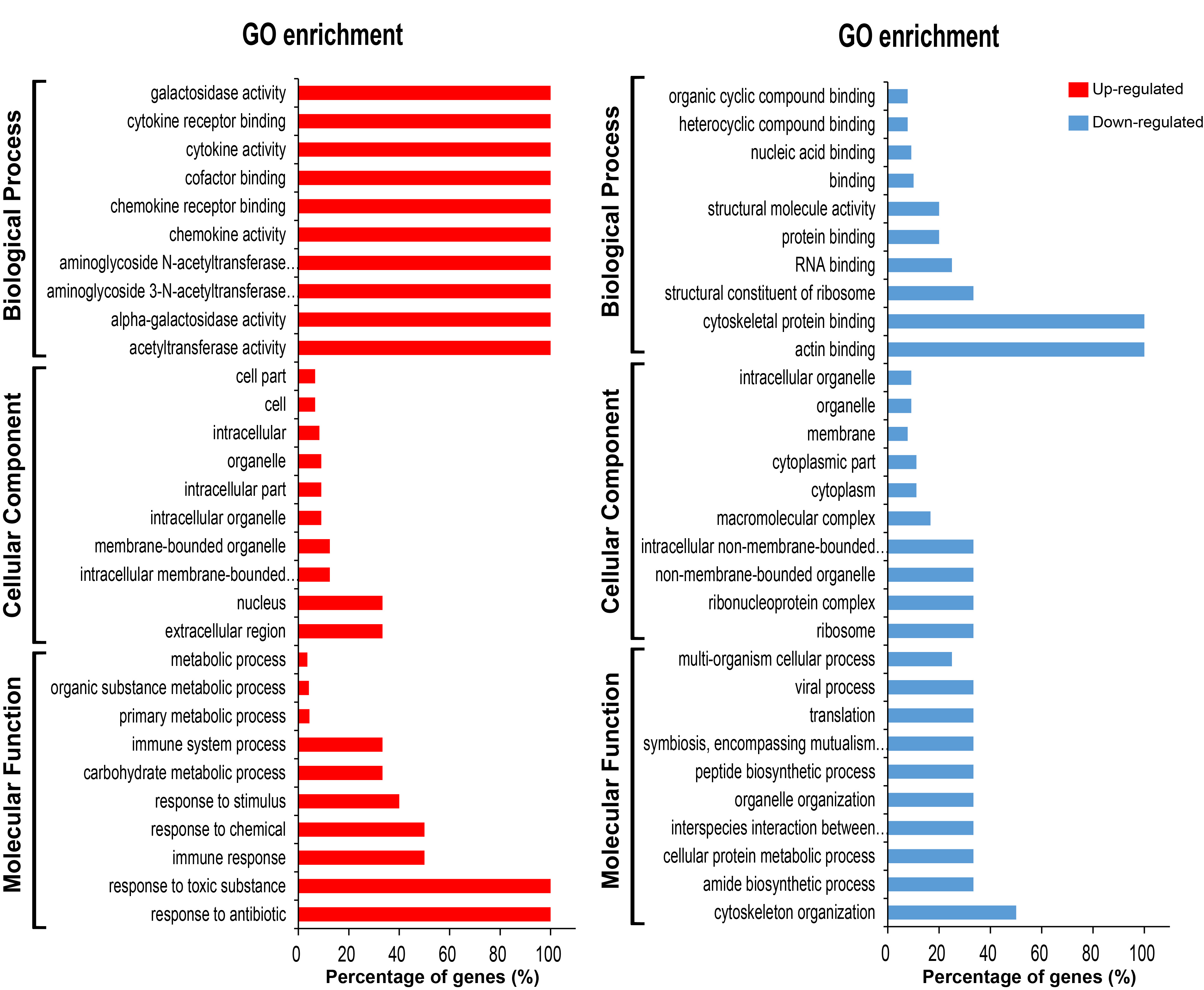

Supplement: Supplementary file 7 — Additional file 7. GO-term enrichments of the differentially expressed lncRNAs. [file 12864_2021_7366_MOESM7_ESM.jpg]

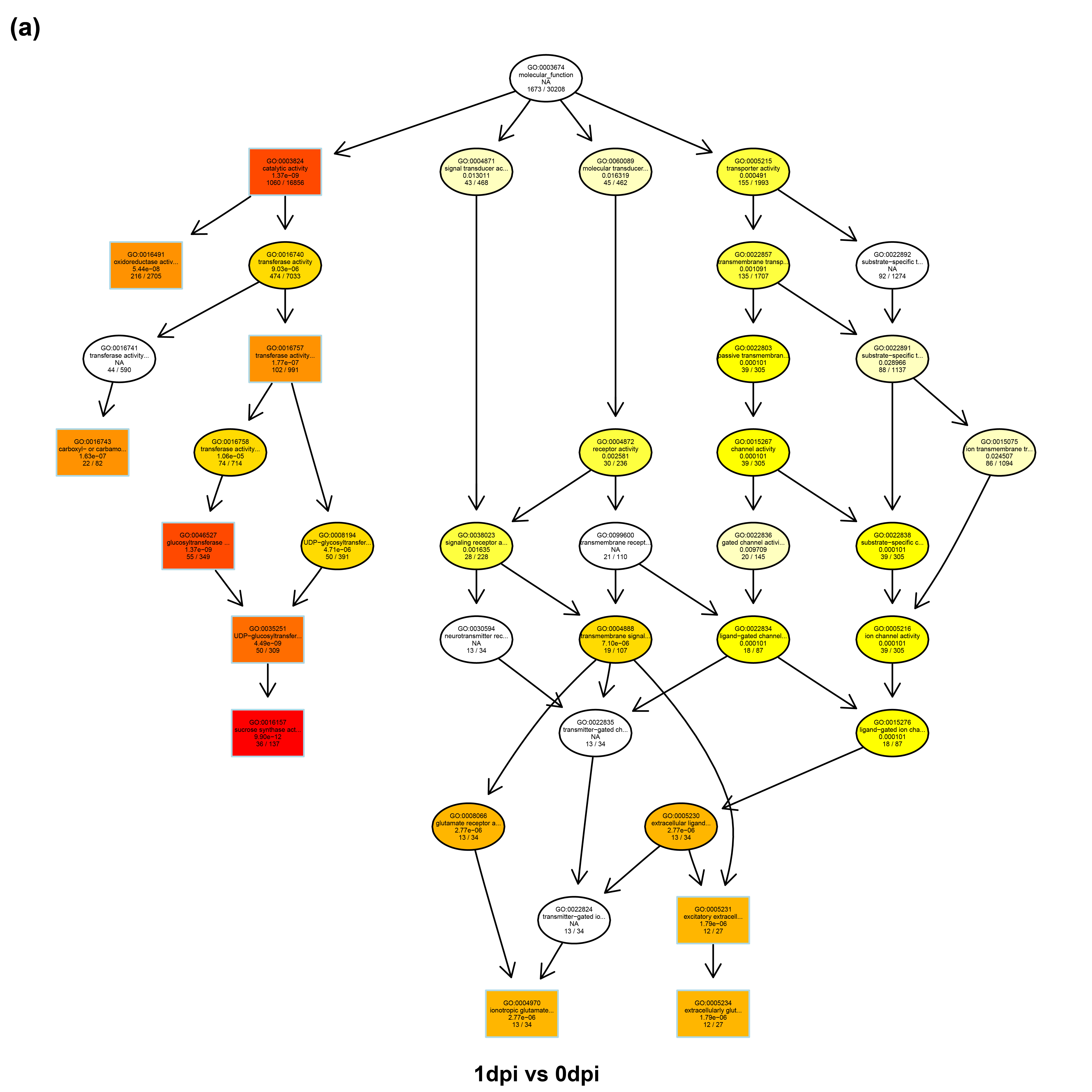

Supplement: Supplementary file 11 — Additional file 11: Directed acyclic graph (DAG) visualization of enriched GO terms for DETs of M. sieversii in response to the V. mali infection at 1 dpi vs 0 dpi. [file 12864_2021_7366_MOESM11_ESM.jpg]

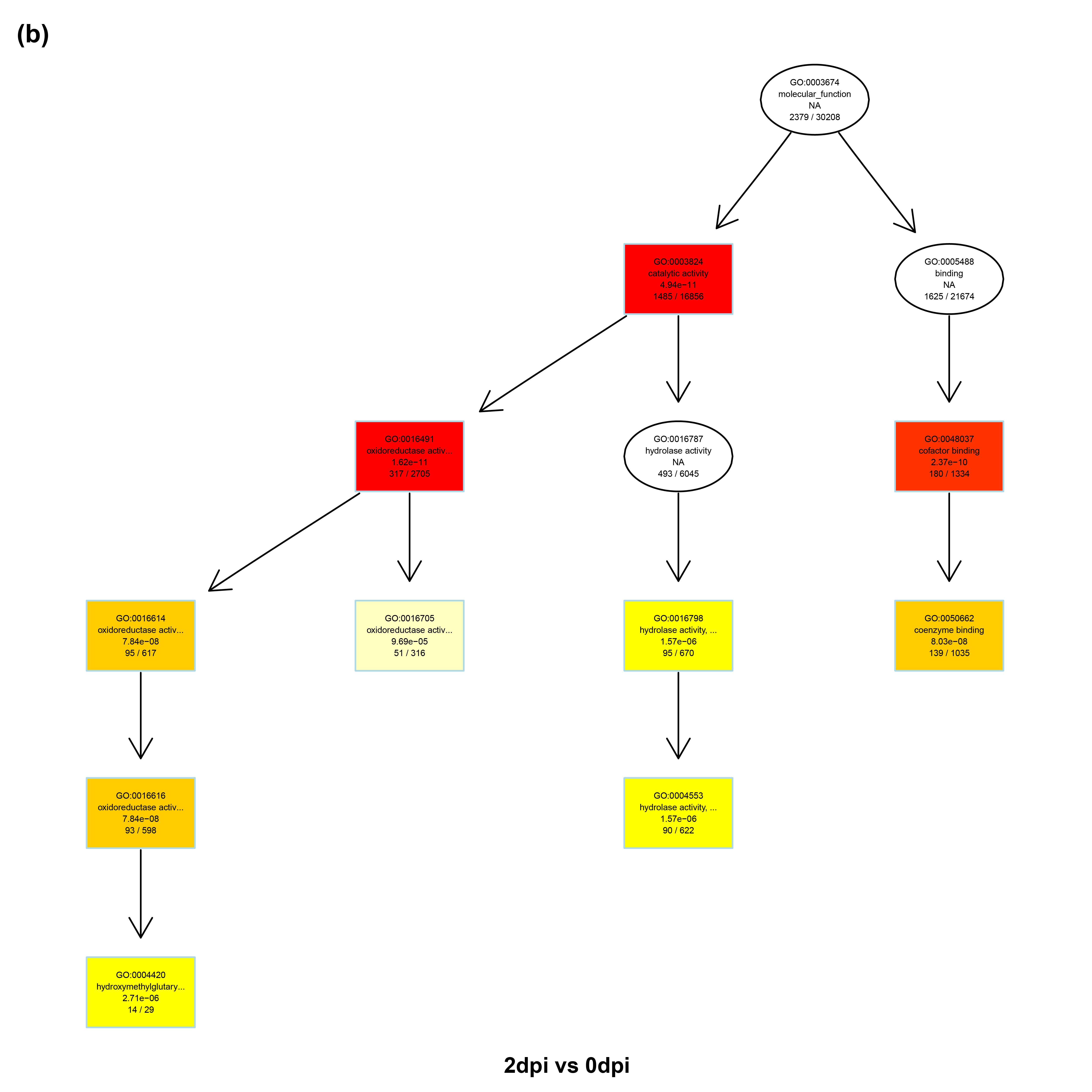

Supplement: Supplementary file 12 — Additional file 12: DAG visualization of enriched GO terms for DETs of M. sieversii in response to the V. mali infection at 2 dpi vs 0 dpi. [file 12864_2021_7366_MOESM12_ESM.jpg]

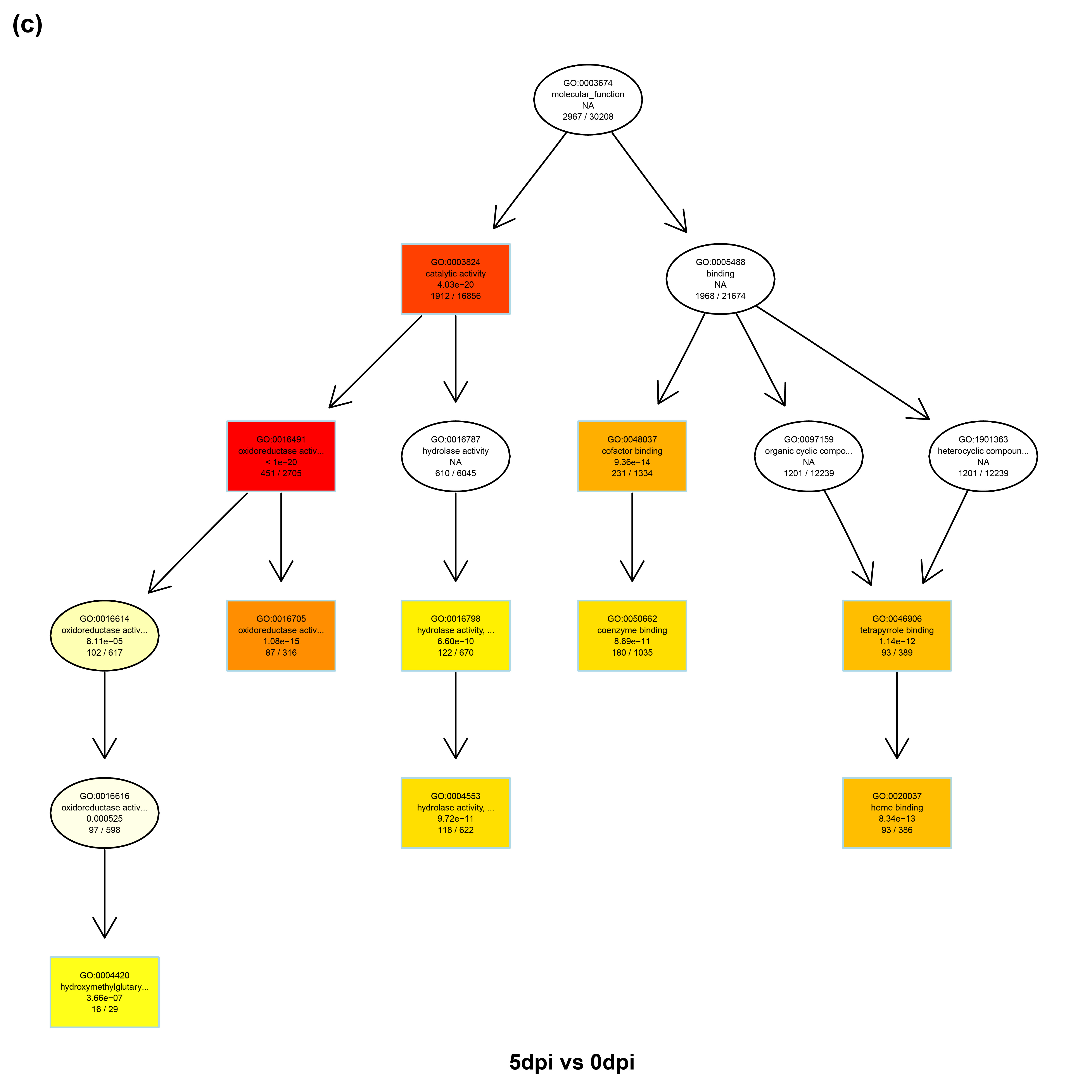

Supplement: Supplementary file 13 — Additional file 13: DAG visualization of enriched GO terms for DETs of M. sieversii in response to the V. mali infection at 5 dpi vs 0 dpi. [file 12864_2021_7366_MOESM13_ESM.jpg]
